# Effect of the introduction of pneumococcal conjugate vaccination on invasive pneumococcal disease in The Gambia: a population-based surveillance study

**DOI:** 10.1016/S1473-3099(16)00054-2

**Published:** 2016-06

**Authors:** Grant A Mackenzie, Philip C Hill, David J Jeffries, Ilias Hossain, Uchendu Uchendu, David Ameh, Malick Ndiaye, Oyedeji Adeyemi, Jayani Pathirana, Yekini Olatunji, Bade Abatan, Bilquees S Muhammad, Augustin E Fombah, Debasish Saha, Ian Plumb, Aliu Akano, Bernard Ebruke, Readon C Ideh, Bankole Kuti, Peter Githua, Emmanuel Olutunde, Ogochukwu Ofordile, Edward Green, Effua Usuf, Henry Badji, Usman N A Ikumapayi, Ahmad Manjang, Rasheed Salaudeen, E David Nsekpong, Sheikh Jarju, Martin Antonio, Sana Sambou, Lamin Ceesay, Yamundow Lowe-Jallow, Momodou Jasseh, Kim Mulholland, Maria Knoll, Orin S Levine, Stephen R Howie, Richard A Adegbola, Brian M Greenwood, Tumani Corrah

**Affiliations:** a Medical Research Council, The Gambia Unit, Atlantic Road, Fajara, The Gambia; b Murdoch Childrens Research Institute, Parkville, Melbourne, Australia; c London School of Hygiene & Tropical Medicine, London, UK; d Centre for International Health, University of Otago, Dunedin, New Zealand; e The National Hospital, Central Area, Garki, Abuja, Nigeria; f Warwick Medical School, University of Warwick, Coventry, UK; g Ministry of Health and Social Welfare, Gambia Government, Kotu, The Gambia; h Bloomberg School of Public Health, Johns Hopkins University, Baltimore, MD, USA; i Department of Paediatrics: Child and Youth Health, University of Auckland, Auckland, New Zealand; j GlaxoSmithKline Vaccines, Wavre, Belgium

## Abstract

**Background:**

Little information is available about the effect of pneumococcal conjugate vaccines (PCVs) in low-income countries. We measured the effect of these vaccines on invasive pneumococcal disease in The Gambia where the 7-valent vaccine (PCV7) was introduced in August, 2009, followed by the 13-valent vaccine (PCV13) in May, 2011.

**Methods:**

We conducted population-based surveillance for invasive pneumococcal disease in individuals aged 2 months and older who were residents of the Basse Health and Demographic Surveillance System (BHDSS) in the Upper River Region, The Gambia, using standardised criteria to identify and investigate patients. Surveillance was done between May, 2008, and December, 2014. We compared the incidence of invasive pneumococcal disease between baseline (May 12, 2008–May 11, 2010) and after the introduction of PCV13 (Jan 1, 2013–Dec 31, 2014), adjusting for changes in case ascertainment over time.

**Findings:**

We investigated 14 650 patients, in whom we identified 320 cases of invasive pneumococcal disease. Compared with baseline, after the introduction of the PCV programme, the incidence of invasive pneumococcal disease decreased by 55% (95% CI 30–71) in the 2–23 months age group, from 253 to 113 per 100 000 population. This decrease was due to an 82% (95% CI 64–91) reduction in serotypes covered by the PCV13 vaccine. In the 2–4 years age group, the incidence of invasive pneumococcal disease decreased by 56% (95% CI 25–75), from 113 to 49 cases per 100 000, with a 68% (95% CI 39–83) reduction in PCV13 serotypes. The incidence of non-PCV13 serotypes in children aged 2–59 months increased by 47% (−21 to 275) from 28 to 41 per 100 000, with a broad range of serotypes. The incidence of non-pneumococcal bacteraemia varied little over time.

**Interpretation:**

The Gambian PCV programme reduced the incidence of invasive pneumococcal disease in children aged 2–59 months by around 55%. Further surveillance is needed to ascertain the maximum effect of the vaccine in the 2–4 years and older age groups, and to monitor serotype replacement. Low-income and middle-income countries that introduce PCV13 can expect substantial reductions in invasive pneumococcal disease.

**Funding:**

GAVI's Pneumococcal vaccines Accelerated Development and Introduction Plan (PneumoADIP), Bill & Melinda Gates Foundation, and the UK Medical Research Council.

## Introduction

In 2008, an estimated 541 000 children younger than 5 years died from pneumococcal disease worldwide.^1^ Africa accounted for 57% of these deaths.^1^ Pneumococcal conjugate vaccines (PCVs) have been effective in high-income countries, reducing the incidence of invasive pneumococcal disease in both vaccinated and unvaccinated individuals.^2^, ^3^, ^4^ 53 low-income countries have now introduced PCV^5^ into their routine immunisation programmes and robust assessment of their effect is a priority.

In South Africa, where the population has a high prevalence of HIV infection, the introduction of the 7-valent PCV (PCV7) was associated with reduced rates of invasive pneumococcal disease in young children and adults.^6^ The Gambia has a high burden of pneumococcal disease and low HIV prevalence.^7^, ^8^ A Gambian trial^9^ of a 9-valent PCV (PCV9), which ended in 2004, showed 77% efficacy against invasive pneumococcal disease caused by vaccine serotypes, 50% against invasive pneumococcal disease overall, 37% against radiological pneumonia, and 16% against overall mortality. Based on these results, and WHO recommendations, the Government of The Gambia introduced PCV7 into the national expanded programme of immunisation (EPI) on Aug 19, 2009, with a schedule of three doses at ages 2, 3, and 4 months, co-administered with the DTPw–HepB–Hib vaccine. In this study, we used standardised population-based surveillance to measure the effect of routine infant vaccination with PCV on invasive pneumococcal disease in The Gambia.

**Research in context**

**Evidence before this study**

We did a systematic literature search of observational studies of the impact of pneumococcal conjugate vaccines (PCVs) on PubMed, Embase, and Web of Science from Jan 1, 2008, to Oct 31, 2015. We searched PubMed using the Medical Subject Headings (MeSH) terms “pneumococcal vaccines”, “vaccines, conjugate”, “meningitis”, “sepsis”, and the (All Fields) terms “pneumococcal”, “conjugate vaccines”, “meningitis”, “sepsis”, “septic(a)emia”, “bacter(a)emia”, “invasive pneumococcal disease”, “impact”, and “effectiveness”. For other data sources, we used the key search terms as above. We searched for population-based studies, published in English language only, of invasive pneumococcal disease that included at least 2 years of data following the introduction of PCV. After reviewing 1504 articles, 33 met inclusion criteria; the impact of PCV7 was measured in 24 studies and that of PCV10 or PCV13 in 13. No published reports were available from low-income countries. Most papers reported laboratory data without ensuring consistency of procedures for screening and investigation of patients. They showed consistent evidence of an overall reduction in invasive pneumococcal disease in children after the introduction of PCV7 (around 50%) with non-vaccine-type disease increasing over time by around 180%. Smaller and more variable reductions (16–48%) occurred in older age groups. Reductions in invasive pneumococcal disease in children after the introduction of PCV10 or PCV13 were consistently around 60–70%, with two of six papers reporting increased non-vaccine type disease and variable reductions in all invasive pneumococcal disease in older age groups.

**Added value of this study**

Our study showed substantial relative and absolute reductions in invasive pneumococcal disease in young children 5 years after the introduction of PCV7 and 3 years following the introduction of PCV13. This study provides robust evidence of the effect of PCV in a low-income country. Our findings are widely relevant because the study was conducted in a typical African population, where the PCV programme used a standard schedule without a catch-up campaign—as is (and will be) the case in almost all low-income and middle-income countries. The study showed that ongoing surveillance is needed to measure the maximum effect of the vaccine in those older than 2 years, wider herd protection effects, vaccine impact against serotype 1, and to monitor serotype replacement. These data are valuable to countries that might need data from a low-income country to support their policy and financing for pneumococcal vaccination.

**Implications of all available evidence**

The routine use of PCV13, with a standard schedule and reasonable coverage, will substantially reduce the burden of invasive pneumococcal disease in low-income and middle-income countries, where the pneumococcal disease burden and associated deaths are highest. Persisting disease due to non-vaccine type pneumococci emphasises the importance of developing new vaccines. Robust data assessing the effect of PCV in Africa are scarce and surveillance must continue in the few sites where high-quality data are being collected.

## Methods

### Study design and participants

This population-based surveillance study was undertaken in Upper River Region, The Gambia, where the UK Medical Research Council has a field station in the town of Basse. Residents of the Basse Health and Demographic Surveillance System (BHDSS) are served by Basse Health Centre and five smaller health facilities (appendix p 16).

We conducted surveillance for all cases of suspected pneumonia, sepsis, and meningitis between May 12, 2008, and Dec 31, 2014. The surveillance population included all residents of the BHDSS aged 2 months or older. The population is enumerated every 4 months, with births, deaths, migrations, and vaccinations recorded. The estimated population in 2014 was 178 510, of whom 32 530 (18%) were children younger than 5 years.

Children younger than 6 months who presented at maternal and child health clinics were eligible to receive all three doses of the vaccine, whereas older children who presented at the clinics were eligible to receive one dose. PCV7 was introduced on Aug 19, 2009, and replaced by PCV13 in May, 2011, without catch-up vaccination.

The study was approved by the Gambia Government–MRC Joint Institutional Ethics Committee (number 1087) and the ethics committee of the London School of Hygiene & Tropical Medicine (London, UK). Participants or their guardians gave written, informed consent.

### Procedures

The surveillance methods used in our population-based study have been described previously.^10^ In brief, nurses assessed all individuals who presented as an outpatient or who were admitted to one of the six health facilities in the study area (Basse, Gambissara, Demba Kunda, Fatoto, Garawol, and Koina). Enrolment involved standardised screening of patients for referral to a clinician in Basse. Clinicians used standardised criteria to identify patients with suspected pneumonia, sepsis, or meningitis, and requested blood culture, lumbar puncture, or chest radiography according to protocol (appendix pp 7–9, 17). Aspiration of pleural fluid of lung aspiration was performed for patients with a pleural effusion or dense peripheral consolidation radiologically. We defined invasive pneumococcal disease as suspected pneumonia, sepsis, or meningitis with isolation. Vaccine failure was defined as invasive pneumococcal disease following two or more doses of PCV covering the homologous serotype, given more than 14 days before the event.^11^, ^12^ Weight was recorded on a digital scale (TANITA, Arlington Heights, IL, USA) and height with a ShorrBoard (Weigh and Measure, Olney, MD, USA). Rapid malaria tests (ICT Diagnostics, Cape Town, South Africa) were done on all patients with suspected pneumonia, sepsis, or meningitis from August to December (the malaria transmission season) each year and in a 10% random sample from January to July each year. This random sample was chosen by random selection of the final digit of the patients' surveillance identity number (0–9) and during the dry season any patient whose identity number ended in zero had a malaria test. Samples were not collected between Oct 5 and Nov 3, 2010, when the field station flooded.

Blood, lung aspirate, cerebrospinal fluid, pleural fluid, and other microbiological samples were processed in Basse using conventional microbiological culture and identification techniques.^13^ *S pneumoniae* was identified by morphology and optochin sensitivity. All pneumococcal isolates were confirmed at the WHO Regional Reference Laboratory (MRC Fajara, The Gambia), and serotyped with a latex agglutination assay using factor and group-specific antisera (Statens Serum Institut, Copenhagen, Denmark). Serotypes 6A and 6B were differentiated from 6C by PCR.^14^ Serotyping of 10% of isolates was repeated at the National Institute for Communicable Diseases in South Africa (Johannesburg, South Africa). The laboratories in Basse and Fajara submitted to external quality assurance throughout the study (UK National External Quality Assessment Service [Sheffield, UK], the WHO Reference Laboratory in Denmark, and the Royal Australasian College of Pathologists [Sydney, Australia]).

### Statistical analysis

The primary outcome of the study was the incidence of invasive pneumococcal disease, in four categories: overall invasive pneumococcal disease; invasive pneumococcal disease caused by PCV7 serotypes (4, 6B, 9V, 14, 18C, 19F, 23F, and cross-reactive 6A);^15^ invasive pneumococcal disease caused by serotypes in PCV13 but not PCV7 (1, 3, 5, 7F, and 19A, excluding serotype 6A); and invasive pneumococcal disease caused by non-vaccine serotypes.

We calculated the incidence of invasive pneumococcal disease by dividing the number of cases by the mid-point population estimates from the BHDSS and multiplying by 100 000. Age groups were prespecified as 2–23 months, 2–4 years, 5–14 years, and 15 years and older (adults). To calculate the incidence in 2008, we extrapolated cases for the unobserved period Jan 1–May 11, 2008. We derived the number of unobserved cases in 2008 by multiplying the number of observed cases from May 12 to Dec 31, 2008, by the average ratio of cases before and after May 12 in each of the years 2009 and 2011–14. The unobserved cases were grouped using the pre-PCV age and serotype distribution from 2008–09. For the flood period in 2010 (Oct 5–Nov 3, 2010), we extrapolated cases using the number of observed cases in 2010 multiplied by the average ratio of cases during the same period in each of the years 2009 and 2011–14. We applied the age and serotype distribution of the observed cases in 2010 to the unobserved cases in 2010.

We corrected for age-specific changes in the number of individuals eligible for investigation per unit population by adjusting the counts of annual invasive pneumococcal disease by age group, assuming the same serotype distribution as that of the observed cases each year. We adjusted annual age-specific counts of invasive pneumococcal disease to the mean rate of enrolment of patients eligible for investigation.

We assessed the effect of the PCV vaccination programme by calculating the ratio of the incidence of invasive pneumococcal disease in the last 2 years of surveillance (2013–14) compared with the baseline first 2 years (May 12, 2008–May 11, 2010). We used the Poisson distribution to calculate incidence rate ratios (IRRs) and 95% CIs. The widths of the confidence intervals were inflated to allow for overdispersion found in the 2–23-month and 5–14-year age groups, estimated from a patient-level Poisson regression analysis of 2008–09 pre-PCV invasive pneumococcal disease data. Statistical significance was set at a p value less than 0·05.

To investigate potential bias caused by temporal changes in health-care seeking, patient investigation, or confounding attributable to secular trends in epidemic serotypes, we did three a-priori stratified analyses, excluding: outpatients, cases identified by lung aspiration alone, and cases caused by serotypes 1 or 5 which exhibit temporal variation in prevalence. To assess the effect of temporal trends in invasive bacterial disease, we analysed the incidence of non-pneumococcal bacteraemia, as a control condition, extrapolating case counts for missing periods in the same manner as for invasive pneumococcal disease. We also analysed temporal changes in the prevalence of contaminated blood cultures, malnutrition, and malaria in patients eligible for investigation.

We used Stata version 12.1 and MATLAB version R2015a for our analyses.

### Role of the funding source

The study was funded by GAVI's Pneumococcal vaccines Accelerated Development and Introduction Plan (PneumoADIP), the Bill & Melinda Gates Foundation, and the UK Medical Research Council. None of the funding sources had any role in collection, analysis, or interpretation of the data. The corresponding author had full access to all the data and was responsible for the final decision to submit for publication.

## Results

**Figure 1:**
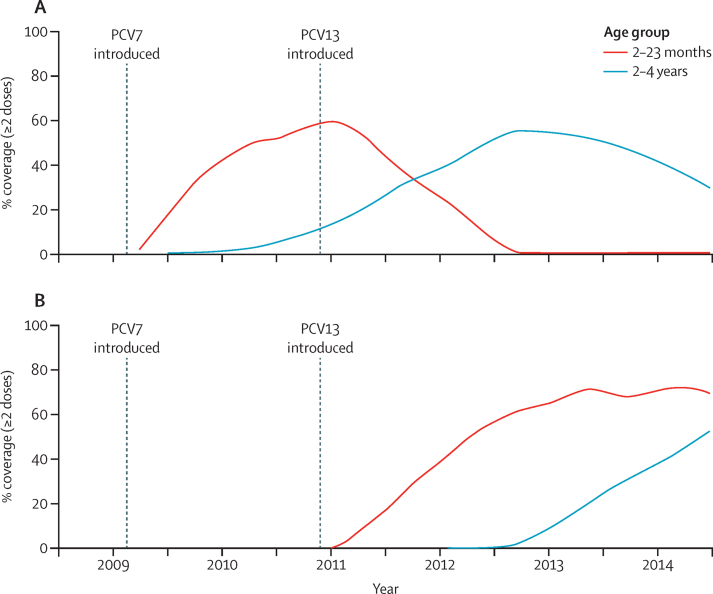
Vaccine coverage Coverage of two or more doses of (A) PCV7 and (B) PCV13, by age group over time.

Our analysis showed that coverage of two or more doses of pneumococcal conjugate vaccine before 12 months of age in children born in the last 6 months of 2013 (indicative of the 2013–14 period) was 94% (3151/3364). Although the first 2 years of surveillance (May 12, 2008–May 11, 2010) overlapped with the introduction of PCV7 (Aug 19, 2009), the coverage of at least two doses of PCV7 in children 2–23 months of age reached only around 35% by April, 2010 (figure 1A) and coverage of one dose in children older than 6 months was only around 4%, indicating that PCV7 had little potential effect in the baseline period. The proportion of children aged 2–23 months who had received at least two doses of PCV13 reached a plateau of around 73% in mid-2013 (figure 1B). The proportion of 2–4-year-old children who had received at least two doses of PCV13 reached 50% by the end of 2014, and was still increasing (figure 1B).

**Figure 2:**
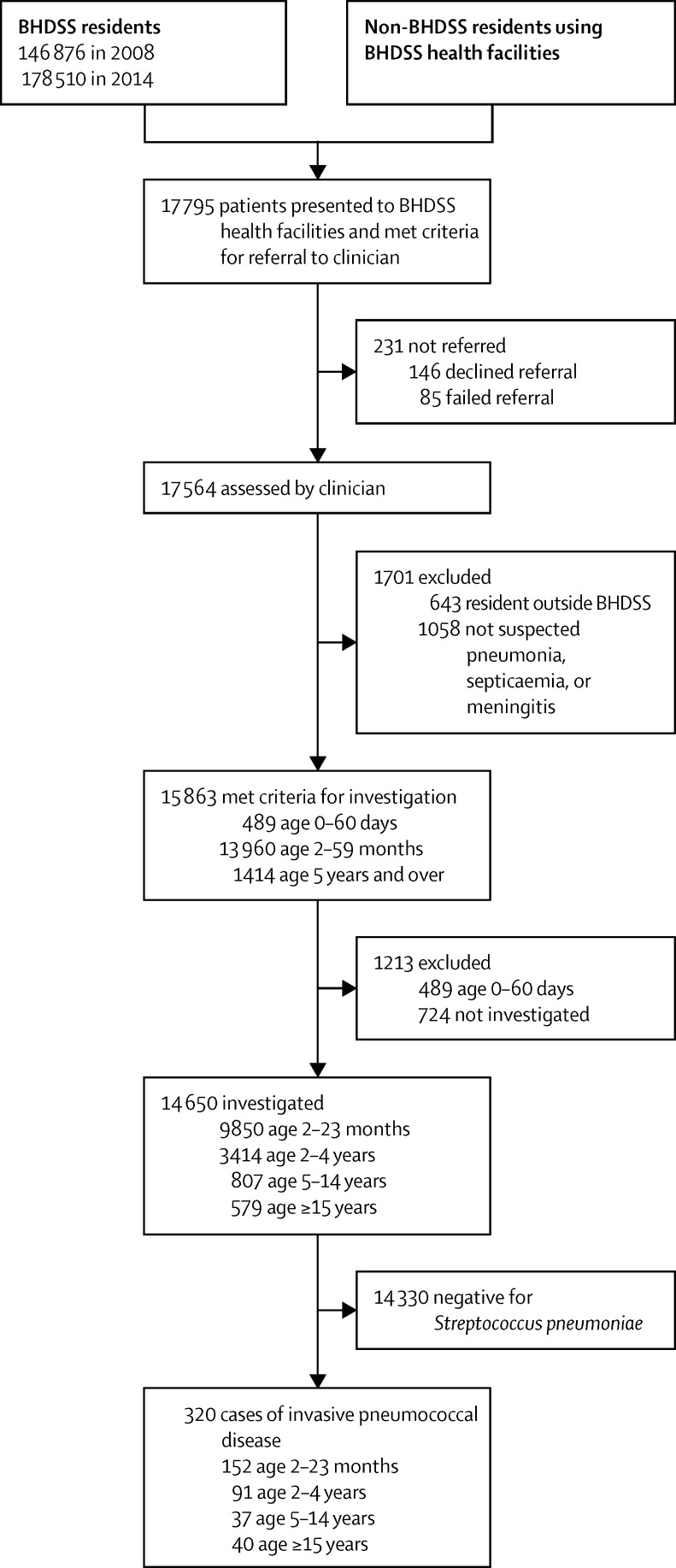
Study profile during the observation period May 12, 2008–Dec 31, 2014 BHDSS=Basse Health and Demographic Surveillance System.

In total, 17 795 patients were screened for referral to a clinician (figure 2). Surveillance performance was consistently high, including the annual proportion of patients referred who were clinically assessed (98–99%), the annual proportion who had microbiological investigation when indicated (90–97%), and the proportion of invasive pneumococcal disease cases with serotyping results (316/320 [99%]). The proportion of patients who had a lung aspiration was roughly 1% in the baseline and 2013–14 periods, and 6% in 2010 and 2011 (appendix p 18).

We identified 320 cases of invasive pneumococcal disease (table 1). Pneumonia was suspected in 212 (66%) of these 320 cases, sepsis in 70 (22%), and meningitis in 38 (12%). 28 (9%) patients died, with a 15% (12/81) mortality rate in the first year of life. Children aged 2–11 months were more likely to have invasive pneumococcal disease caused by non-vaccine serotypes than were those aged 1–4 years (51/79 [65%] *vs* 33/160 [21%], p<0·0009). There were no significant differences in sex (p=0·790), nutritional status (p=0·090), or mortality (p=0·122) between vaccine and non-vaccine serotype cases.

**Figure 3:**
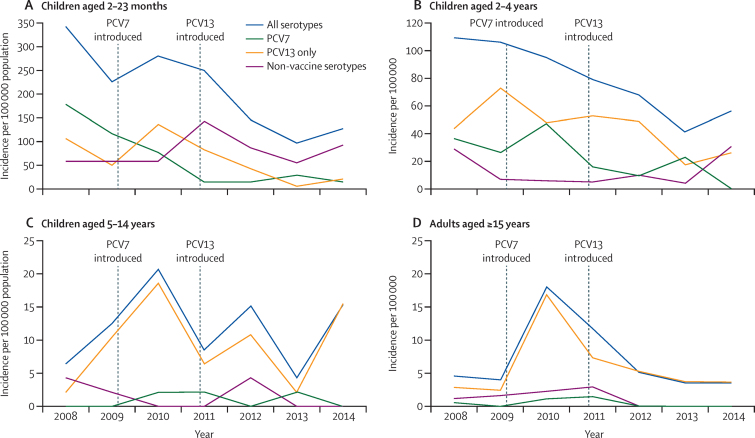
Adjusted annual incidence of invasive pneumococcal disease from 2008 to 2014, by age group and serotype (A) Children aged 2–23 months. (B) Children aged 2–4 years. (C) Children aged 5–14 years. (D) Adults aged ≥15 years. 320 cases of invasive pneumococcal disease occurred in total; in six there were two different serotypes detected in different samples and in four the isolate could not be serotyped. The adjustment corrects for trends in the enrolment of patients eligible for investigation.

Across all age groups, 111 cases of invasive pneumococcal disease were recorded in the baseline 2 years (2008–10); 38 (34%) were PCV7 serotype, 55 (50%) were PCV13-only serotype, and 21 (26%) were non-vaccine type (table 2). In the 2013–14 period, there were 67 cases of invasive pneumococcal disease across all age groups; 12 (18%) were PCV7 serotype, 26 (38%) were PCV13-only serotype, and 29 (44%) were non-vaccine type. After adjustment of case counts, there was a 55% (95% CI 30–71) reduction in the incidence of all invasive pneumococcal disease from baseline to 2013–14 in children aged 2–23 months, from 253 cases per 100 000 population to 113 cases per 100 000 (table 2, figure 3). The incidence of invasive pneumococcal disease also fell by 56% (95% CI 25–75) in children aged 2–4 years, from 113 to 49 cases per 100 000. In children aged 5–14 years, there was a non-significant reduction of invasive pneumococcal disease of 16% (95% CI −125 to 69). The incidence in adults (aged ≥15 years) also fell, albeit non-significantly, by 59% (95% CI −3 to 84), from nine to four cases per 100 000.

In the 2–23 months age group, PCV7-type invasive pneumococcal disease fell by 83% (95% CI 57–93), from a baseline of 122 cases per 100 000 to 21 per 100 000 in 2013–14; PCV13-type invasive pneumococcal disease fell by 82% (64–91), from 195 to 35 cases per 100 000; and PCV13-only type invasive pneumococcal disease also decreased by 82% (44–94), from 78 to 14 cases per 100 000. Analysis restricted to the 6–23 months age group, with vaccine coverage higher than 80% (appendix p 19), is shown in appendix p 10. Smaller reductions occurred in 2–4-year-old children: PCV7-type invasive pneumococcal disease fell by 74% (95% CI 26–91) from 44 to 11 cases per 100 000, PCV13-type disease fell by 68% (39–83) from 99 to 31 cases per 100 000, and PCV13 only-type disease fell by 62% (15–83) from 58 to 22 per 100 000 (table 2, figure 3). There was no evidence of a reduction in PCV13 or PCV13-only-type invasive pneumococcal disease in the 5–14-year-old age group. In adults (aged ≥15 years), there were some reductions in the incidence of PCV13-type invasive pneumococcal disease (50% [95% CI −32 to 81]) and PCV13-only-type invasive pneumococcal disease (48% [–39 to 80]), although these decreases were not significant.

We noted a non-significant increase of 48% (95% CI −30 to 213) in non-vaccine-type invasive pneumococcal disease in the 2–23 months age group, from 49 to 75 cases per 100 000, and of 27% (95% CI −61 to 313) in 2–4-year-olds. Overall, non-vaccine-type invasive pneumococcal disease increased by 47% (−21 to 175) in infants aged 2–59 months.

The estimates of the effect of PCVs were unchanged in stratified analyses that excluded outpatients and cases detected only by lung aspiration (appendix pp 11–12). When cases caused by serotypes 1 or 5 were excluded, the effect of vaccine against PCV13 serotypes was unchanged (appendix p 13). The incidence of the control condition of non-pneumococcal bacteraemia did not change between the baseline years and the 2013–14 period (appendix pp 14, 20).

The prevalence of malaria in patients aged 2–59 months and those aged 5 years and older, who were eligible for investigation, fluctuated between 5% and 17%, and 10% and 24% respectively, throughout the study period, with no significant difference in prevalence between baseline and 2013–14 (appendix pp 21–22). Throughout the study, in children eligible for investigation, the prevalence of malnutrition remained stable at around 14% (appendix p 23), as was the prevalence of blood culture contamination at around 7% (appendix p 24).

**Figure 4:**
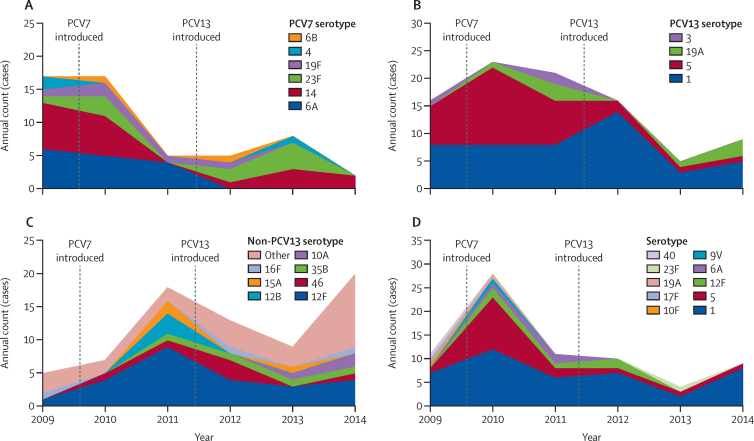
Annual counts of serotype-specific invasive pneumococcal disease (A) PCV7 serotypes in children aged 2–59 months. (B) PCV13-only serotypes in children aged 2–59 months. (C) Non-PCV13 serotypes in children aged 2–59 months. Serotypes are shown separately if three or more episodes. Other serotypes (peach colour) with two episodes: 2, 9A, 13, 17F, 24F, 25F, and 33F; one episode: 9N, 10F, 11B, 12A, 15B, 18A, 20, 21, 22A, 25A, and 40. (D) All serotypes in individuals aged 5 years and older.

In terms of serotype-specific changes, the effect of PCV7 vaccination in the 2–59-month age group was evident from 2011 onwards, with reduced cases of serotypes 6A and 14 (figure 4A). Cases of serotype 5 were reduced in 2013–14 (figure 4B). 15 different serotypes contributed to the increase in non-vaccine-type invasive pneumococcal disease in 2014 (figure 4C). In the 5 years and older age group, a peak number of serotype 5 cases occurred in 2010, with one case detected in 2013 and one in 2014 (figure 4D). The number of serotype 1 episodes in 2014 was similar to that reported in earlier years of surveillance (figure 4D). 17 episodes of invasive pneumococcal disease were associated with vaccine failure; two each for serotypes 1, 6A, 19A, and 19F; four for 23F; and five for serotype 14. Four children with vaccine failure were severely malnourished (appendix p 15).

## Discussion

Population-based surveillance for almost 7 years in The Gambia has shown that, in the 2–23 months age group, following the introduction of PCVs into the EPI, there was an 82% reduction in invasive pneumococcal disease caused by PCV13 serotypes and a 55% reduction in all invasive pneumococcal disease. In the 2–4 years age group, there was a 68% reduction in invasive pneumococcal disease caused by PCV13 serotypes and a 56% reduction in all types of invasive pneumococcal disease.

Our findings are similar to those reported in other settings soon after implementation of PCV. Estimates of the effect of PCV7 on all invasive pneumococcal disease in children younger than 2 years have varied between 56% in England and Wales^16^ and 69% in USA^15^ and South Africa,^6^ with a somewhat greater effect associated with PCV10 or PCV13 (62% in Oxford in the UK,^17^ 71% in Denmark,^18^ 78% in England and Wales,^4^ and 80% in Finland).^19^ Our study might have underestimated vaccine impact in the 2–23 months age group because of the possible effect of PCV7 in our baseline period and the fact that the proportion of children with at least two doses of this vaccine had not peaked by the beginning of 2013. The 56% reduction in invasive pneumococcal disease in the 2–4 years age group recorded in our study is less than the 75% reported in England and Wales,^4^, ^16^ although our finding is probably an underestimate.

Herd protection in unimmunised children and adults following the introduction of PCV has been documented in many settings.^4^, ^6^, ^15^, ^16^, ^17^, ^18^ Consistent with these findings, we noted a trend towards reduced invasive pneumococcal disease in adults, but our findings should be interpreted with caution given the small numbers of cases. Data from Kilifi, Kenya, suggest likely herd protection effects of PCV10, since carriage of vaccine-type pneumococci in children and older individuals was reduced by two-thirds following an extensive catch-up campaign.^20^

We observed a 48% (95% CI −30 to 213) increase in non-PCV13 type invasive pneumococcal disease in the 2–59 months age group, although this increase was not significant. Following the introduction of PCV7 in developed countries, non-vaccine-type invasive pneumococcal disease increased by two to three fold, although this increase was largely caused by serotypes covered by the PCV13 vaccine.^21^ Non-vaccine-type invasive pneumococcal disease has increased following the introduction of PCV13 in Denmark,^18^ and England and Wales,^4^ but such a change is not yet evident in the USA.^22^ Several years of surveillance post-introduction of PCV13 might be necessary to assess the full effect.^21^

We recorded temporal changes in some serotypes that were probably independent of vaccine introduction. Serotype 5 peaked in all age groups in 2010, whereas an increase in serotype 12F disease occurred in children younger than 5 years in 2011 (figure 4). Such temporal changes in serotypes, and the low numbers of cases, necessitate cautious interpretation of serotype-specific results. The effect of PCV13 against serotype 1 that has been reported in other settings^4^, ^6^, ^23^ was not yet evident in our study. Effects against this important serotype might need further time for the coverage of PCV13 to increase in the 2–4 years age group in which serotype 1 is more prevalent than in the first 2 years of life. Continued surveillance is needed to confirm the effect of vaccination against this serotype in our setting.

Our study has several strengths. All screening, clinical investigation criteria, case definitions, and laboratory practices were standardised and applied consistently throughout the study.^10^ However, there were some limitations. After initial piloting, we did only 16 months of surveillance before the introduction of PCV7. However, the proportion of children who had received at least two doses of PCV7 remained low for several months after vaccine introduction, allowing a 2-year baseline period. Before and after studies are prone to bias and confounding caused by changes in factors apart from vaccination that affect the detection and risk of pneumococcal disease in the population. Our analysis provided some reassurance in this regard, with adjustment for temporal changes in the rate of patient investigation, demonstration of a stable incidence of the control condition of non-pneumococcal bacteraemia, and no change in estimates in stratified analyses.

The effect of vaccination that we observed in The Gambia resulted from a PCV programme using a standard schedule and an introduction with effectively no catch-up campaign, which are the programmatic circumstances that exist in most low-income countries. Thus, our findings have important implications for EPI programmes in other low-income countries and provide reassurance for those that have already introduced PCV. Questions to answer going forward that make ongoing surveillance in The Gambia and a few other settings a high priority include measuring the maximum effect in those older than 2 years, the extent of herd protection, the magnitude of serotype replacement, and the effect on pneumonia. These data will provide crucial information regarding considerations of alternative immunisation schedules and the need and prioritisation of modified conjugate vaccines and vaccines designed to prevent disease caused by all pneumococcal serotypes.

## Figures and Tables

**Table 1:** Characteristics of cases of invasive pneumococcal disease between May 12, 2008, and Dec 31, 2014, stratified by serotype

		**All invasive pneumococcal disease (n=320)**	PCV7-type disease (n=69)*	PCV13-type disease only (n=158)*	Non-vaccine-type disease (n=95)*
Age group					
	2–11 months	81 (25%)	15 (22%)	13 (8%)	51 (54%)
	12–23 months	71 (22%)	23 (33%)	32 (20%)	17 (18%)
	2–4 years	91 (29%)	25 (37%)	51 (33%)	16 (17%)
	5–14 years	37 (12%)	3 (4%)	30 (19%)	4 (4%)
	≥15 years	40 (13%)	3 (4%)	32 (20%)	7 (7%)
Sex					
	Male patients	194 (61%)	40 (58%)	99 (62%)	55 (58%)
	Female patients	126 (39%)	29 (42%)	59 (38%)	40 (42%)
Diagnostic category†					
	Meningitis	38 (12%)	11 (16%)	10 (6%)	17 (18%)
	Sepsis	70 (22%)	8 (12%)	29 (18%)	32 (34%)
	Pneumonia	212 (66%)	50 (72%)	119 (76%)	46 (48%)
Pathology					
	Proven meningitis	23 (7%)	8 (12%)	3 (2%)	12 (13%)
	Non-meningitis	297 (93%)	61 (88%)	155 (98%)	83 (87%)
Weight-for-height *Z* score <–3 (age 2–59 months)		39/243 (16%)	13/63 (21%)	10/96 (10%)	16/84 (19%)
Treated as inpatient		291 (91%)	63 (91%)	146 (92%)	84 (88%)
Mortality		28 (9%)	7 (10%)	9 (6%)	13 (14%)
	Proven meningitis	8 (3%)	4 (6%)	0	4 (4%)
	Non-meningitis	20 (6%)	3 (4%)	9 (6%)	9 (10%)

PCV7=serotypes covered by the 7-valent pneumococcal conjugate vaccine: 4, 6B, 9V, 14, 18C, 19F, 23F, and cross-reactive 6A. PCV13 only=serotypes covered only by the 13-valent pneumococcal conjugate vaccine: 1, 3, 5, 7F, and 19A. Non-vaccine type=serotypes not covered by PCV13.

* The number of episodes of PCV7, PCV13 only, and non-vaccine type invasive pneumococcal disease do not add up to 320 cases; in six cases two different serotypes were detected in different samples and in four cases the isolate could not be serotyped.

† Categories of surveillance diagnosis (appendix p 8) are mutually exclusive and ranked by severity; meningitis is the most severe, then sepsis, and then pneumonia.

**Table 2:** Crude and adjusted numbers of cases and incidence of invasive pneumococcal disease in the baseline period (May 12, 2008–May 11, 2010), and in the 2013–14 period post-vaccine introduction and incidence rate ratios, by age group and serotype

	**May, 2008–April, 2010 adjusted (crude) cases**	**May, 2008–April, 2010 adjusted (crude) incidence per 100 000 population**	**2013–14 adjusted (crude) cases**	**2013–14 adjusted (crude) incidence per 100 000 population**	**Crude incidence rate ratio 2013–14 *vs* May, 2008–April, 2010 (95% CI)**	**Adjusted incidence rate ratio 2013–14 *vs* May, 2008–April, 2010 (95% CI)**
**Age 2–23 months**						
All	55 (48)	253 (220)	32 (32)	113 (114)	0·52 (0·33–0·82)	0·45 (0·29–0·70)
PCV7	27 (23)	122 (106)	6 (6)	21 (21)	0·21 (0·08–0·51)	0·17 (0·07–0·43)
PCV13	43 (37)	195 (170)	10 (10)	35 (36)	0·21 (0·10–0·43)	0·18 (0·09–0·36)
PCV13 only	17 (15)	78 (69)	4 (4)	14 (14)	0·21 (0·07–0·64)	0·18 (0·06–0·56)
Non-vaccine type	11 (11)	49 (50)	21 (21)	75 (75)	1·48 (0·70–3·13)	1·48 (0·70–3·13)
**Age 2–4 years**						
All	36 (33)	113 (102)	20 (22)	49 (55)	0·53 (0·31–0·91)	0·44 (0·25–0·75)
PCV7	14 (13)	44 (40)	5 (5)	11 (12)	0·31 (0·11–0·86)	0·26 (0·09–0·74)
PCV13	32 (29)	99 (90)	13 (14)	31 (35)	0·39 (0·20–0·73)	0·32 (0·17–0·61)
PCV13 only	19 (17)	58 (53)	9 (10)	22 (25)	0·47 (0·21–1·02)	0·38 (0·17–0·85)
Non-vaccine type	5 (4)	14 (12)	7 (8)	18 (20)	1·60 (0·48–5·30)	1·27 (0·39–4·13)
**Age 5–14 years**						
All	10 (11)	12 (13)	10 (9)	10 (9)	0·69 (0·25–1·85)	0·84 (0·31–2·25)
PCV7	0 (0)	Unspecified	1 (1)	1 (1)	Unspecified	Unspecified
PCV13	8 (9)	10 (11)	10 (9)	10 (9)	0·84 (0·30–2·38)	1·05 (0·37–2·99)
PCV13 only	8 (9)	10 (11)	9 (8)	9 (8)	0·75 (0·26–2·18)	0·95 (0·32–2·76)
Non-vaccine type	2 (2)	2 (2)	0	Unspecified	Unspecified	Unspecified
**Age ≥15 years**						
All	14 (19)	9 (12)	7 (4)	4 (2)	0·18 (0·06–0·54)	0·41 (0·16–1·03)
PCV7	1 (2)	1 (1)	0	Unspecified	Unspecified	Unspecified
PCV13	11 (15)	7 (9)	7 (4)	4 (2)	0·23 (0·08–0·70)	0·50 (0·19–1·32)
PCV13 only	11 (14)	7 (9)	7 (4)	4 (2)	0·25 (0·08–0·76)	0·52 (0·20–1·39)
Non-vaccine type	3 (4)	2 (2)	0	Unspecified	Unspecified	Unspecified

PCV7=serotypes covered by PCV7: 4, 6B, 9V, 14, 18C, 19F, 23F, and cross-reactive 6A. PCV13=serotypes covered by PCV13. PCV13 only=serotypes covered by PCV13 but not PCV7: 1, 3, 5, 7F, and 19A. Non-vaccine type=serotypes not covered by PCV13. In the 2–23 months age group, in the baseline period there were 48 cases of invasive pneumococcal disease and 49 episodes of serotype-specific invasive pneumococcal disease. In the 2–4 years age group, in the baseline period there were 33 cases of invasive pneumococcal disease and 34 episodes of serotype-specific invasive pneumococcal disease, and in 2013–14 there were 22 cases of invasive pneumococcal disease and 23 episodes of serotype-specific invasive pneumococcal disease. In the ≥15 years age group, in the baseline period there were 19 cases of invasive pneumococcal disease and 20 serotype-specific episodes. Case counts are adjusted for trends in patients eligible for investigation and rounded to the nearest integer. 95% CIs were calculated taking into account overdispersed Poisson distributions in the 2–23 months and 5–14 years age groups.
