# Supplementary material for: Effect of the introduction of pneumococcal conjugate vaccination on invasive pneumococcal disease in The Gambia: a population-based surveillance study
Source: Lancet Infect Dis. 2016 Jun;16(6):703–11. doi: 10.1016/S1473-3099(16)00054-2 (PMC4909992; doi:10.1016/S1473-3099(16)00054-2)
Supplement: Supplementary appendix [file mmc1.pdf]

THE LANCET Infectious Diseases

Supplementary webappendix

This webappendix formed part of the original submission and has been peer reviewed. We post it as supplied by the authors.

Supplement to: Mackenzie GA, Hill PC, Jeffries DJ, et al. Effect of the introduction of pneumococcal conjugate vaccination on invasive pneumococcal disease in The Gambia: a population-based surveillance study. *Lancet Infect Dis* 2016; published online Feb 17. http://dx.doi.org/10.1016/S1473-3099(16)00054-2.

Webappendix

Impact of the introduction of pneumococcal conjugate vaccination on invasive pneumococcal disease in The Gambia: population-based surveillance before and after vaccine introduction

Supplementary methods

1. Surveillance methods and procedures

The objective of this study was to determine the impact of pneumococcal conjugate vaccine (PCV) on the incidence of pneumococcal disease at the population level.

1.1 Study setting

Clinical surveillance was conducted at all health facilities in the area of the Basse Health and Demographic Surveillance System (BHDSS) which covers a population of approximately 180,000 on the south bank of the River Gambia. The health needs of this population are served by the Basse Health Centre, which receives referrals from five smaller health facilities, Gambisara, Demba Kunda, Fatoto, Garawol, and Koina. Maternal-child-health teams provided immunisation services at three base clinics and 34 outreach sites. In 2009, the estimated infant and under-5 year mortality rates in the BHDSS were 33 and 62 per 1,000 live births respectively. HIV prevalence among antenatal women in the area is around 1.0%.[1]

1.2 Surveillance population

The BHDSS, which covers an area of 1111 square kilometres, has been in operation since 2007. All residents of the BHDSS aged 2 months and greater were included in the study. Residence was defined as continual residence within the BHDSS for 4 or more months as determined at 4-monthly visits to all households. The estimated population in 2008 was 146 876, increasing to 179 108 in 2014. Although individuals who are not resident in the BHDSS do present to the health facilities in the area, this analysis includes only individuals who were confirmed resident at the time of illness. Residence was confirmed by the individual having been enumerated in the BHDSS population listing. Individuals enrolled in surveillance who are not found in the BHDSS population listing were visited at their household in order to ascertain residential status; if confirmed as residents they were enumerated through BHDSS procedures.

1.3 Case ascertainment

Patients were eligible for recruitment if they presented as an outpatient, or were admitted to one of the six health facilities in the BHDSS between May 12, 2008 and December 31, 2014. Patients who presented to any of these health facilities were screened for referral to a clinician by

designated surveillance staff using standardised criteria (Table S1). Clinicians assessed patients referred to them using standardised diagnostic criteria (Table S2). A diagnosis of suspected pneumonia, septicaemia, and/or meningitis led to standardised investigations (Table S3). Chest radiographs were taken on all cases of suspected pneumonia and if the attending clinician judged the investigation beneficial to patient management. Blood cultures were performed for all cases of suspected pneumonia, sepsis, or meningitis. Lumbar puncture was undertaken in cases of suspected meningitis. Lung aspiration was undertaken in selected cases of pneumonia when the following criteria were met: a) a radiographic area of dense, peripheral, pneumonic consolidation, b) stable respiratory status, and c) written informed consent was provided by the patient or parent or guardian of the participant child.

Blood was collected for culture using a sterile technique and inoculated into a culture bottle (Bactec Peds Plus, Bactec Anaerobic, Bactec Aerobic, Becton Dickinson; or conventional tryptone soy and brain heart infusion bottles for a minority of samples collected at night in outlying clinics). The weight of blood culture bottles was measured before and after sample collection. Lung aspirates were transported immediately to the laboratory and inoculated onto agar and examined using Gram stain. Cerebrospinal fluid, pleural fluid, and other microbiological samples were processed consistently using standard methods.[2]

Radiographs were obtained using a portable system (HF-110A, DynaRad, Illinois, USA) with a consistent radiographic technique and digital processing (CR 120/140, Kodak, New York, USA). Procedures to produce digital images were in accordance with WHO recommendations.[3] Two readings of each radiograph (masked to identity and date) were undertaken by three independent readers and discordant results were resolved by a paediatric radiologist. All readers were calibrated to the WHO standard.[3] All readers were required to achieve very high levels of agreement with blinded samples of the WHO standard set of 222 radiographs (kappa statistic >0.8) before they read the study radiographs. Assessment of the radiologic pneumonia endpoint will compare the pre-PCV period (2008/09) with the 13PCV period (2013/14). The results of the impact of PCV on the incidence of radiologic pneumonia will be presented in a subsequent paper following completion of data collection and analysis.

Mortality in the BHDSS was monitored during 4-monthly visits to all households. We defined mortality as death of a resident recorded in the BHDSS database. Trained field workers administer standardised verbal autopsy questionnaires to respondents concerning all deaths in the BHDSS area. Possible pneumococcal deaths were defined as deaths of individuals with a verbal autopsy categorised as pneumonia, sepsis, or meningitis by the Inter-VA algorithm.[4] As complete data for all-cause and possible pneumococcal mortality are not yet available this analysis is being deferred until data are complete. The process of recording all deaths in the BHDSS in 2014 will be completed at the end of 2015. Assessment of the mortality endpoints will compare the pre-PCV period (2008/09) with the 13PCV period (2013/14). All-cause mortality will be stratified, including and excluding the malaria season, which will remove some bias due to possible changes in mortality due to malaria. The results of the impact of PCV on mortality will be presented in a subsequent paper following the completion of data collection and analysis.

1.4 Case definition

IPD was defined as an illness clinically compatible with this diagnosis accompanied by isolation of *S. pneumoniae* from a normally sterile site. We classified IPD as being caused by a) PCV7 vaccine serotypes: 4, 6B, 9V, 14, 18C, 19F, 23F, and 6A due to demonstrated cross-protection,[5] b) PCV13-non-PCV7 vaccine serotypes: 1, 3, 5, 7F, and 19A, and c) non-PCV13 vaccine serotypes being all other types. Non-typeable isolates were excluded. Episodes were considered as separate events if the first and subsequent consultations were at least 30 days apart, or if pneumococcus of a different serotype was isolated during each episode. Events were defined as radiologic pneumonia if the criteria described section 1.3 were fulfilled and if the patient had a suspected diagnosis of pneumonia and the radiologic appearance and procedure for classification was according to the WHO standard.[3]

1.5 Laboratory methods

An automated system (Bactec 9050, Becton Dickinson, UK) was used for blood cultures. Bottles that signalled positive were sub-cultured onto blood agar, chocolate agar, and McConkey agar. Bottles which failed to signal within 5 days were considered negative. Isolates grown on these sub-cultures were identified using conventional microbiological techniques and biochemical tests (API, Biomerieux). *S. pneumoniae* was identified by colony morphology, susceptibility to ethylhydrocupreine and, if susceptibility was equivocal, by bile solubility, and reaction with polyvalent antisera (Statens Serum Institut, Copenhagen, Denmark). Isolates classified as contaminants included coagulase-negative staphylococcus, bacillus species, micrococcus species, and *Streptococcus viridans*.

Pneumococcal isolates were transported to the MRC Fajara laboratory for serotyping using a latex agglutination assay which employs factor and group-specific antisera (Statens Serum Institut, Copenhagen, Denmark). Serotypes 6A and 6B were differentiated from 6C by polymerase chain reaction using a 6C-specific DNA primer (CDC, Atlanta, USA). Molecular serotyping was repeated for 10% of isolates (CDC, Atlanta, USA) and external quality control serotyping was performed at the National Institute for Infectious Diseases in South Africa for 10% of isolates and all isolates of serogroups 6 or 9. The laboratories in Basse and Fajara submitted to external quality assurance programmes throughout the study (UK National External Quality Assessment Service, WHO reference laboratory Denmark, Royal Australasian College of Pathologists).

2. Surveillance time periods for the before and after analysis

Piloting of surveillance procedures was undertaken between September 2007 and May 2008. The baseline period was from May 12, 2008 until May 11, 2010. The vaccine period was from January 1, 2013 until December 31, 2013.

3. Before and after statistical analysis

3.1 Vaccination

In order to illustrate the rate of uptake of PCV in different age groups included in the analysis, we plotted the coverage over time of two or more doses of PCV7 and PCV13 in the 2-23 months and 2-4 years age groups. To demonstrate vaccine coverage during the 2013/14 period which was used for comparison with the baseline period, we calculated the coverage of two or more

doses of PCV before 12 months of age in a cohort of BHDSS residents born in the second half of 2013.

3.2 Population denominator

Annual incidence was calculated using BHDSS estimates of the age-specific mid-point population. Comparisons of before and after incidence used weighted averages of the mid-point populations in the respective age-strata and annual time periods, May 12, 2008–May 11, 2010 and 2013/2014.

3.3 Definition of events

Cases of IPD in which two different serotypes were isolated were classified as two different episodes of serotype-specific IPD if the two different serotypes belonged to different serotype categories.

3.4 Extrapolation of case counts in 2008 and 2010

We extrapolated the number of unobserved cases between January 1 and May 11, 2008 and also during the flood period between October 5 and November 3, 2010. These extrapolated cases were used for plots of annual incidence over time but not used in calculations of incidence rate ratios.

The expected number of cases from January 1 to May 11, 2008 was the product of the number of cases between May 12 and December 31, 2008 and the average of the ratios of annual cases occurring during January 1 to May 11 compared to May 12 to December 31 in 2009 and 2011-2014. The age and serotype distribution of the extrapolated cases was the same as the age and serotype distribution of observed cases in 2008 and 2009.

Unobserved cases in 2010 were extrapolated for the 30 days of interrupted IPD surveillance. The expected number of unobserved cases was the product of the number of observed cases in 2010 and the average of the annual ratios of cases which occurred between October 5 and November 3 in 2009 and 2011-2014. The age and serotype distribution of the extrapolated cases was the same as for the observed cases in 2010.

3.5 Adjustment of annual case counts

The number of children enrolled in surveillance and eligible for investigation per unit population increased over time, while the rate of enrolment of older children and adults eligible for investigation decreased. We corrected for these changes in the sensitivity of case ascertainment adjusting the crude counts of annual IPD cases, by age group, assuming the serotype distribution was the same as that of the observed cases each year. Annual, age-specific counts of IPD were adjusted using the difference between the mean rate of enrolment of patients eligible for investigation during the study period and smoothed curves fitted to plots of annual rates over time. The peak of enrolment of children eligible for investigation occurred in 2011 and 2012 and our adjustment reduced the number of cases in these two years and increased the number of cases in 2008, 2009, 2013, and 2014. Thus, a result of our adjustment of annual case counts was that the number of IPD cases in children that are included in the incidence rate ratio calculations, comparing the first and final two years of surveillance, are slightly greater in the adjusted analysis than in the crude analysis.

3.6 Incidence analysis

Incidence was defined as the number of cases divided by the mid-point population at risk. Vaccine impact was calculated as the difference in age-specific incidence subtracting the incidence after vaccination from the incidence before the introduction of PCV,

$$IRD = Incidence\ before\ 7PCV - Incidence\ after\ 7PCV$$

The incidence rate ratio (IRR) was calculated as the ratio of incidence after to before PCV, introduction indicating vaccine effectiveness at the population level. Vaccine effectiveness was calculated as (1-IRR) x 100.

$$IRR = Incidence\ after\ 7PCV / Incidence\ before\ 7PCV$$

The IRR analysis was based on a Poisson distribution. We assessed the validity of this distributional assumption by modelling the age-specific incidence of IPD using pre-PCV data from 2008–2009. Our assessment of distributional validity indicated the Poisson distribution was acceptable for the 2-4 years and ≥15 years age groups. We used overdispersed Poisson distributions for the 2-23 months and 5-14 years age groups with variance inflation factors of 1.057 and 1.268 respectively.

3.7 Bias and confounding

To investigate potential bias due to temporal changes in health care seeking behaviour, patient investigation, or confounding due secular trends in epidemic serotypes we conducted *a priori* stratified analyses excluding outpatients, cases identified by lung aspiration alone, and cases caused by serotype 1 or 5. The IRR estimates using the crude data and data excluding outpatients, cases identified by lung aspirate alone, and cases of serotype 1 or 5 were compared and if they differed by greater than 20% we concluded that clinically significant confounding was present. We conducted these stratified analyses of the IRR using crude data and data with adjusted case counts accounting for changes over time in the sensitivity of case ascertainment (section 3.5). We determined the adjusted, stratified, and age-specific annual case counts by multiplying the adjusted count by the proportion of IPD cases treated as an outpatient and detected by lung aspiration alone, in the baseline and PCV13 periods and by age strata, assuming an unchanging serotype distribution. The adjusted data excluding cases of serotype 1 and 5 used the same approach as for excluding outpatients but also accounted for the proportion of IPD due to serotype 1 and 5 in the baseline and PCV13 periods and within age strata.

To assess the effect of temporal trends in invasive bacterial disease, we evaluated the crude and adjusted incidence of non-pneumococcal bacteraemia obtained by the surveillance system, as a control condition. Non-pneumococcal bacteraemia was defined as a positive blood culture in which the isolate was not a contaminant, *S. pneumoniae*, *Serratia liquefaciens*, or *N. meningitidis*. *S. liquefaciens* was not included as we experienced a nosocomial epidemic of this bacterium in the latter half of 2010. Meningococcal isolates were not included in non-pneumococcal bacteraemia because of the epidemic nature of meningococcal disease; in fact, the surveillance area experienced an epidemic of meningococcal disease in 2012.[8] Non-pneumococcal bacteraemia was detected using the same procedures as for detection of IPD and it is unlikely to be influenced by PCV. Therefore, the incidence of non-pneumococcal bacteraemia is a robust control condition indicating potential changes in the sensitivity of surveillance and/or

changes in population risk factors for invasive bacterial disease and IPD which are unrelated to PCV. Annual counts of non-pneumococcal bacteraemia were extrapolated in 2008 and 2010 in same manner as for IPD. We also adjusted the annual and comparison period counts of non-pneumococcal bacteraemia in order to plot incidence over time and evaluate the IRR.

In our setting, significant risk factors for IPD are malnutrition and malaria. Therefore, we also evaluated whether there were any changes over time in the prevalence of malnutrition or malaria in patients who were enrolled in surveillance and eligible for investigation.

Reference List

(1) National AIDS Control Programme. Republic of The Gambia, HIV Sentinel Surveillance Report 2011. 1 May 2012. Banjul, Ministry of Health & Social Welfare.

(2) Adegbola RA, Falade AG, Sam BE, et al. The etiology of pneumonia in malnourished and well-nourished Gambian children. *Pediatr Infect Dis J* 1994;**13**:975-82.

(3) World Health Organization Pneumonia Vaccine Trial Investigators' Group. Standardization of interpretation of chest radiographs for the diagnosis of pneumonia in children. World Health Organization, Department of Vaccines and Biologicals 2001. http://www.who.int/vaccines-documents (accessed October 3, 2004).

(4) Garenne M. Prospects for automated diagnosis of verbal autopsies. *BMC Med* 2014;**12**:18.

(5) Whitney CG, Pilishvili T, Farley MM et al. Effectiveness of seven-valent pneumococcal conjugate vaccine against invasive pneumococcal disease: a matched case-control study. *Lancet* 2006;**368**:1495-502.

(6) Cutts FT, Zaman SM, Enwere G et al. Efficacy of nine-valent pneumococcal conjugate vaccine against pneumonia and invasive pneumococcal disease in The Gambia: randomised, double-blind, placebo-controlled trial. *Lancet* 2005;**365**:1139-1146.

(7) World Health Organization. *Pocket book of hospital care for children: guidelines for the management of common illnesses with limited resources*. 1st ed. Geneva: World Health Organization, 2005.

(8) Hossain MJ, Roca A, Mackenzie G et al. Serotype W135 meningococcal disease, The Gambia, 2012. *Emerg Infect Dis* 2013;**19**:1507-10.

Supplementary Tables

Table S1. Screening criteria for referral of out- and in-patients for clinician assessment (if one or more criteria are present for 14 days or less)

Patients aged 2 to 59 months

- History of cough or difficulty breathing, plus raised respiratory rate for age
- Lower chest wall indrawing, nasal flaring, or grunting
- Oxygen saturation less than 92%
- History of convulsion
- Impaired consciousness*
- Bulging fontanelle
- Stiff neck
- Axillary temperature at least 38°C, or less than 36°C, in a patient admitted or being admitted
- Prostration†
- Weight below -3 z score for age
- Local musculoskeletal swelling or tenderness
- **Irrespective of age or residential location, any child with possible meningitis**

Patients aged 5 years or greater

- History of cough and difficulty breathing or pleuritic chest pain or supraclavicular/sternal recession or nasal flaring
- History of productive cough and fever
- History of convulsion
- Impaired consciousness *
- Altered mental state
- Photophobia
- Neck stiffness
- Axillary temperature of at least 38°C, or less than 36°C, in a patient admitted or being admitted
- History of rigors
- Local musculoskeletal swelling or tenderness
- **Irrespective of age or residential location, any patient with possible meningitis**

*Impaired consciousness is defined as V, P, or U on the AVPU score, where A is if the patient is alert, V if responsive to verbal stimulus, P if responsive to pain stimulus, and U if unresponsive. †Prostration is defined as an inability to drink or breast feed, or to remain in a seated position in a child otherwise able to do so.

Table S2. Clinical definitions for suspected pneumonia, septicaemia, and meningitis

	Age ≥2 months and <5 years	Age ≥5 years
**Suspected pneumonia**	Suspected pneumonia is defined if there is a history of cough or difficulty breathing of less than 14 days' duration, accompanied by one or more of:<br>1. Raised respiratory rate for age*<br>2. Lower chest wall indrawing, nasal flaring or grunting<br>3. Oxygen saturation less than 92%<br>4. Focal chest signs (dull percussion note, coarse crackles, bronchial breathing)	Suspected pneumonia will be defined according to clinical judgement and is to be considered in patients presenting with an illness of 14 days' duration or less, if two or more of the following are present::<br>1. Cough<br>2. Haemoptysis<br>3. Pleuritic chest pain<br>4. Breathlessness<br>5. Axillary temperature ≥38°C
**Suspected meningitis**	Suspected meningitis will be defined according to clinical judgement and is to be considered if any of the following are present:<br>1. Neck stiffness<br>2. Impaired consciousness†<br>3. Prostration‡<br>4. History of convulsion<br>5. Bulging fontanelle	Suspected meningitis will be defined according to clinical judgement and is to be considered if two or more of the following are present:<br>1. Axillary temperature ≥38°C<br>2. Meningism (neck stiffness and/or photophobia)<br>3. Altered mental state (Glasgow Coma Score <14)
**Suspected septicaemia**	Suspected septicaemia will be defined as one or more of:<br>1. Clinician diagnosis of focal sepsis (including but not limited to: septic arthritis, osteomyelitis, endocarditis, peritonitis, liver abscess, soft tissue abscess, cellulitis)<br>2. For a patient admitted, or being admitted, axillary temperature is <36°C or ≥38°C and no obvious cause of fever<br>3. For a patient admitted, or being admitted, the clinical impression is of severe malnutrition§	Suspected septicaemia will be defined as one or more of:<br>1. Clinician diagnosis of focal sepsis (including but not limited to: septic arthritis, osteomyelitis, endocarditis, peritonitis, liver abscess, soft tissue abscess, cellulitis)<br>2. For a patient admitted, or being admitted, axillary temperature is <36°C or ≥38°C and no obvious cause of fever<br>3. History of rigors

*Raised respiratory rate for age is defined as greater than 50 breaths per minute for children at least 2 months but less than 12 months, and as greater than 40 breaths per minute for children at least 12 months but less than 60 months.

†Impaired consciousness is defined as V, P, or U on the AVPU score, where A is if the patient is alert, V if responsive to verbal stimulus, P if responsive to pain stimulus, and U if unresponsive.

‡Prostration is defined as inability to drink or breast feed, or to remain in a seated position in a child otherwise able to do so.

§Severe malnutrition is defined according to the WHO definition.[7]

Table S3. Guideline for investigation of patients referred to clinicians and diagnosed with suspected pneumococcal disease according to clinical definitions

a. Patients with suspected pneumococcal disease are to have blood culture.

b. Patients with suspected meningitis are to have lumbar puncture.

c. Patients with suspected pneumonia are to have chest X-ray.

d. Chest X-ray should also be considered in patients with meningitis or septicaemia if the clinician's impression is of co-existing pneumonia or if it is judged that a chest X-ray will assist in management.

e. Lung aspirate should be considered for a patient if peripheral consolidation has been demonstrated, preferably by X-ray.

f. Other investigations including pleural tap and joint aspirate may be considered according to the clinical indication.

g. Patients with suspected pneumococcal disease are to have,

    i. a rapid diagnostic test for malaria (January – July; only if surveillance number ends in '0').

    ii. serum collection for antibiotic activity detection if surveillance number ends in '0' or '5' and the patient is enrolled in Basse.

Table S4. Crude and adjusted number of cases and incidence of IPD in the 6-23 month age group in the baseline period of May 12, 2008 to May 11, 2010 and in the 2013/14 period post-vaccine introduction, by serotype

	5/2008–4/2010 adjusted (crude) cases	5/2008–4/2010 adjusted (crude) incidence per 100 000	2013/14 adjusted (crude) cases	2013/14 adjusted (crude) incidence per 100 000	Crude incidence rate ratio 2013/14 relative to 5/2008–4/2010 (95% CI)	Adjusted incidence rate ratio 2013/14 relative to 5/2008–4/2010 (95% CI)
**Age 6-23 months**						
All	46 (40)	286 (249)	24 (24)	116 (116)	0·46 (0·28–0·78)	0·40 (0·24–0·67)
PCV7	22 (19)	137 (118)	6 (6)	29 (29)	0·24 (0·10–0·63)	0·21 (0·08–0·53)
PCV13	36 (31)	224 (193)	8 (8)	39 (39)	0·20 (0·09–0·44)	0·17 (0·08–0·38)
PCV13 only	15 (13)	93 (81)	2 (2)	10 (10)	0·12 (0·03–0·55)	0·10 (0·03–0·47)
Non-vaccine type	10 (9)	62 (56)	15 (15)	72 (72)	1·29 (0·55–3·02)	1·16 (0·51–2·64)

Table S5. Crude and adjusted number of cases and incidence of IPD, excluding cases identified by lung aspiration alone, in the baseline period of May 12, 2008 to May 11, 2010 and in the 2013/14 period post-vaccine introduction, by age group and serotype

	5/2008–4/2010 adjusted (crude) cases	5/2008–4/2010 adjusted (crude) incidence per 100 000	2013/14 adjusted (crude) cases	2013/14 adjusted (crude) incidence per 100 000	Crude incidence rate ratio 2013/14 relative to 5/2008–4/2010 (95% CI)	Adjusted incidence rate ratio 2013/14 relative to 5/2008–4/2010 (95% CI)
**Age 2-23 months**						
All	49 (43)	227 (197)	28 (28)	99 (99)	0·50 (0·31–0·82)	0·44 (0·27–0·71)
PCV7	24 (21)	109 (96)	5 (6)	18 (21)	0·22 (0·09–0·56)	0·16 (0·06–0·43)
PCV13	38 (34)	175 (156)	9 (9)	31 (32)	0·20 (0·10–0·44)	0·18 (0·09–0·39)
PCV13 only	15 (14)	70 (64)	4 (3)	13 (11)	0·17 (0·05–0·60)	0·21 (0·07–0·64)
Non-vaccine type	9 (9)	44 (41)	18 (19)	65 (67)	1·63 (0·72–3·69)	1·55 (0·70–3·52)
**Age 2-4 years**						
All	33 (30)	103 (93)	18 (20)	45 (50)	0·53 (0·30–0·94)	0·44 (0·25–0·77)
PCV7	13 (13)	40 (40)	4 (5)	10 (12)	0·31 (0·11–0·86)	0·26 (0·08–0·77)
PCV13	29 (26)	90 (81)	12 (13)	29 (32)	0·40 (0·20–0·78)	0·31 (0·16–0·63)
PCV13 only	17 (14)	53 (43)	8 (9)	20 (22)	0·51 (0·22–1·18)	0·38 (0·17–0·88)
Non-vaccine type	4 (4)	13 (12)	7 (7)	16 (17)	1·40 (0·41–4·77)	1·27 (0·37–4·37)
**Age 5-14 years**						
All	8 (9)	10 (11)	9 (8)	9 (8)	0·75 (0·26–2·18)	0·95 (0·32–2·76)
PCV7	0 (0)	unsp.	1 (1)	unsp.	unsp.	unsp.
PCV13	7 (7)	8 (8)	9 (8)	9 (8)	0·96 (0·31–3·01)	1·08 (0·36–3·28)
PCV13 only	7 (7)	8 (8)	8 (7)	8 (7)	0·84 (0·26–2·73)	0·96 (0·31–3·01)
Non-vaccine type	1 (2)	2 (2)	0 (0)	unsp.	unsp.	unsp.
**Age ≥15 years**						
All	11 (17)	7 (11)	7 (4)	4 (2)	0·21 (0·07–0·61)	0·50 (0·19–1·32)
PCV7	1 (2)	1 (1)	0 (0)	unsp.	unsp.	unsp.
PCV13	9 (13)	6 (8)	7 (4)	4 (2)	0·27 (0·09–0·83)	0·62 (0·23–1·70)
PCV13 only	9 (12)	5 (7)	7 (4)	4 (2)	0·29 (0·09–0·91)	0·65 (0·23–1·78)
Non-vaccine type	2 (4)	1 (2)	0 (0)	unsp.	unsp.	unsp.

PCV7=serotypes covered by PCV7. PCV13=serotypes covered by PCV13. PCV13 only=serotypes covered by PCV13 but not PCV7. Non-vaccine type=serotypes not covered by PCV13. Exclusion of cases from adjusted counts used the age- and year-specific distributions of IPD cases identified by lung aspiration alone. Adjusted case counts are rounded to the nearest integer. Confidence intervals calculated taking into account overdispersed Poisson distributions in the 2–23 months and 5–14 years age groups.

Table S6. Crude and adjusted number of cases and incidence of IPD, excluding cases treated as outpatients, in the baseline period of May 12, 2008 to May 11, 2010 and in the 2013/14 period post-vaccine introduction, by age group and serotype

	5/2008–4/2010 adjusted (crude) cases	5/2008–4/2010 adjusted (crude) incidence per 100 000	2013/14 adjusted (crude) cases	2013/14 adjusted (crude) incidence per 100 000	Crude incidence rate ratio 2013/14 relative to 5/2008–4/2010 (95% CI)	Adjusted incidence rate ratio 2013/14 relative to 5/2008–4/2010 (95% CI)
**Age 2-23 months**						
All	51 (44)	232 (202)	29 (29)	103 (103)	0·51 (0·31–0·83)	0·44 (0·28–0·70)
PCV7	24 (22)	112 (101)	5 (5)	19 (18)	0·18 (0·06–0·48)	0·16 (0·06–0·43)
PCV13	39 (35)	179 (161)	9 (9)	32 (32)	0·20 (0·09–0·42)	0·18 (0·08–0·38)
PCV13 only	16 (14)	72 (64)	4 (4)	13 (14)	0·22 (0·07–0·69)	0·19 (0·06–0·60)
Non-vaccine type	10 (9)	45 (41)	19 (19)	68 (67)	1·63 (0·72–3·69)	1·47 (0·67–3·23)
**Age 2-4 years**						
All	31 (28)	96 (87)	18 (20)	45 (50)	0·57 (0·32–1·01)	0·47 (0·26–0·83)
PCV7	12 (11)	37 (34)	4 (5)	10 (12)	0·36 (0·13–1·04)	0·27 (0·09–0·84)
PCV13	27 (25)	84 (78)	12 (13)	29 (32)	0·41 (0·21–0·81)	0·34 (0·17–0·68)
PCV13 only	16 (15)	49 (47)	8 (9)	20 (22)	0·48 (0·21–1·09)	0·41 (0·18–0·96)
Non-vaccine type	4 (3)	12 (9)	7 (7)	16 (17)	1·86 (0·48–7·20)	1·36 (0·39–4·82)
**Age 5-14 years**						
All	8 (9)	10 (11)	10 (9)	10 (9)	0·84 (0·30–2·38)	1·05 (0·37–2·99)
PCV7	0 (0)	unsp.	1 (1)	1 (1)	unsp.	unsp.
PCV13	7 (8)	8 (9)	10 (9)	10 (9)	0·95 (0·32–2·76)	1·20 (0·40–3·56)
PCV13 only	7 (8)	8 (9)	9 (8)	9 (8)	0·84 (0·28–2·53)	1·08 (0·36–3·28)
Non-vaccine type	1 (1)	2 (2)	0 (0)	unsp.	unsp.	unsp.
**Age ≥15 years**						
All	9 (16)	6 (10)	7 (3)	4 (2)	0·16 (0·05–0·56)	0·65 (0·23–1·78)
PCV7	1 (2)	1 (1)	0 (0)	unsp.	unsp	unsp.
PCV13	7 (13)	4 (8)	7 (3)	4 (2)	0·20 (0·06–0·71)	0·80 (0·28–2·31)
PCV13 only	7 (12)	4 (7)	7 (3)	4 (2)	0·22 (0·06–0·78)	0·83 (0·28–2·43)
Non-vaccine type	2 (3)	1 (2)	0 (0)	unsp.	unsp.	unsp.

PCV7=serotypes covered by PCV7. PCV13=serotypes covered by PCV13. PCV13 only=serotypes covered by PCV13 but not PCV7. Non-vaccine type=serotypes not covered by PCV13. Exclusion of cases from adjusted counts used the age- and year-specific distributions of outpatient cases of IPD. Adjusted case counts are rounded to the nearest integer. Confidence intervals calculated taking into account overdispersed Poisson distributions in the 2–23 months and 5–14 years age groups.

Table S7. Crude and adjusted number of cases and incidence of IPD, excluding cases of serotype 1 or 5, in the baseline period of May 12, 2008 to May 11, 2010 and in the 2013/14 period post-vaccine introduction, by age group and serotype

	5/2008–4/2010 adjusted (crude) cases	5/2008–4/2010 adjusted (crude) incidence per 100 000	2013/14 adjusted (crude) cases	2013/14 adjusted (crude) incidence per 100 000	Crude incidence rate ratio 2013/14 relative to 5/2008–4/2010 (95% CI)	Adjusted incidence rate ratio 2013/14 relative to 5/2008–4/2010 (95% CI)
**Age 2-23 months**						
All	41 (36)	190 (165)	29 (29)	103 (103)	0·62 (0·38–1·03)	0·55 (0·34–0·89)
PCV7	27 (23)	122 (106)	6 (6)	21 (21)	0·21 (0·08–0·53)	0·17 (0·07–0·43)
PCV13	29 (25)	133 (115)	7 (7)	25 (25)	0·23 (0·09–0·54)	0·19 (0·08–0·44)
PCV13 only	2 (2)	10 (9)	1 (1)	4 (4)	0·39 (0·03–4·56)	0·39 (0·03–4·56)
Non-vaccine type	11 (11)	49 (50)	21 (21)	75 (75)	1·48 (0·70–3·13)	1·48 (0·70–3·13)
**Age 2-4 years**						
All	20 (19)	62 (59)	14 (16)	36 (40)	0·67 (0·35–1·31)	0·58 (0·30–1·15)
PCV7	14 (13)	44 (40)	5 (5)	11 (12)	0·31 (0·11–0·86)	0·26 (0·09–0·74)
PCV13	17 (15)	51 (47)	6 (7)	16 (17)	0·37 (0·15–0·91)	0·30 (0·12–0·76)
PCV13 only	2 (2)	7 (6)	3 (3)	7 (7)	1·20 (0·20–7·16)	0·96 (0·16–5·64)
Non-vaccine type	5 (4)	14 (12)	7 (8)	18 (20)	1·60 (0·48–5·30)	1·27 (0·39–4·13)
**Age 5-14 years**						
All	2 (2)	2 (2)	1 (1)	1 (<1)	0·42 (0·03–6·27)	0·42 (0·03–6·27)
PCV7	0 (0)	unsp.	1 (1)	1 (<1)	unsp.	unsp.
PCV13	0 (0)	unsp.	0 (1)	1 (<1)	unsp.	unsp.
PCV13 only	0 (0)	unsp.	0 (0)	unsp.	unsp.	unsp.
Non-vaccine type	2 (2)	2 (2)	0 (0)	unsp.	unsp.	unsp.
**Age ≥15 years**						
All	4 (6)	3 (4)	0 (0)	unsp.	unsp.	unsp.
PCV7	1 (2)	<1 (1)	0 (0)	unsp.	unsp.	unsp.
PCV13	1 (2)	<1 (1)	0 (0)	unsp.	unsp.	unsp.
PCV13 only	0 (0)	unsp.	0 (0)	unsp.	unsp.	unsp.
Non-vaccine type	3 (4)	2 (2)	0 (0)	unsp.	unsp.	unsp.

PCV7=serotypes covered by PCV7. PCV13=serotypes covered by PCV13. PCV13 only=serotypes covered by PCV13 but not PCV7. Non-vaccine type=serotypes not covered by PCV13. Exclusion of cases from adjusted counts used the age-, year-, and serotype category-specific distributions of IPD case due to serotypes 1 or 5. Adjusted case counts are rounded to the nearest integer. Confidence intervals calculated taking into account overdispersed Poisson distributions in the 2–23 months and 5–14 years age groups.

Table S8. Crude and adjusted number of cases and incidence of non-pneumococcal bacteraemia in the baseline period of May 12, 2008 to May 11, 2010 and the 2013/14 period post-vaccine introduction, by age group

Age group	5/2008–4/2010 adjusted (crude) cases	5/2008–4/2010 adjusted (crude) incidence per 100 000	2013/14 adjusted (crude) cases	2013/14 adjusted (crude) incidence per 100 000	Crude incidence rate ratio 2013/14 relative to 5/2008–4/2010 (95% CI)	Adjusted incidence rate ratio 2013/14 relative to 5/2008–4/2010 (95% CI)
2–23 months	50 (43)	230 (197)	69 (69)	244 (245)	1·24 (0·85–1·82)	1·07 (0·73–1·55)
2–4 years	11 (10)	35 (31)	18 (20)	45 (50)	1·60 (0·75–3·41)	1·29 (0·61–2·72)
5–14 years	5 (5)	5 (6)	8 (7)	8 (7)	1·18 (0·37–3·71)	1·34 (0·38–4·73)
≥15 years	4 (7)	2 (4)	2 (1)	1 (<1)	0·13 (0·02–1·02)	0·38 (0·06–2·39)

Adjusted case counts take account of changes over time in the sensitivity of case ascertainment, and are rounded to the nearest integer. Confidence intervals calculated taking account of overdispersed Poisson distributions in the 2–23 months and 5–14 years age groups. Numbers of individual bacteraemia cases throughout the observation period: *E. coli* (n=31), *Acinetobacter* sp. (n=4), *H. influenzae* type b (n=16), *H. influenzae* non-type b (n=11), *Klebsiella* sp. (n=13), Non-typhi salmonella (n=39), *Staphylococcus aureus* (n=62), *Pseudomonas* sp. (n=17), Other gram positive (n=23), Other gram negative (n=49)

Table S9. Characteristics of children with invasive pneumococcal disease and vaccine failure

Count	Date onset of illness	Age (mo)	Suspected diagnosis	Weight-for-Height z-score <-3	Child HIV infection	Mother HIV infection	Serotype	Clinical sample	Vaccine	No. doses prior to illness	No. months since last dose
1	27 May 2010	14	Pneumonia	Yes	ND	ND	19F	LA	PCV7	3	5
2	15 June 2010	12	Pneumonia	No	ND	ND	14	LA	PCV7	3	7
3	11 Nov 2010	15	Pneumonia	No	ND	ND	6A	LA & BC	PCV7	3	8
4	7 May 2012 D	16	Pneumonia	Yes	ND	ND	19F	BC	PCV7	3	11
5	2 Dec 2012	25	Meningitis	No	ND	ND	23F	BC	PCV7	3	7
6	8 Jan 2013	27	Meningitis	No	ND	ND	23F	CSF & BC	PCV7	2	18
7	2 Feb 2013	32	Pneumonia	No	No	No	23F	BC	PCV7	3	26
8	11 Feb 2013	16	Pneumonia	No	Yes	Yes	14	BC	PCV13	3	10
9	25 Feb 2013	20	Sepsis	No	ND	ND	23F	BC	PCV13	2	12
10	14 Mar 2013	30	Pneumonia	No	No	ND	1/6A/B	BC	PCV7	3	23
11	4 Aug 2013	18	Pneumonia	No	No	No	1	LA	PCV13	3	11
12	5 Aug 2013	11	Pneumonia	No	No	ND	14	BC	PCV13	3	8
13	26 Aug 2013	19	Pneumonia	No	No	ND	19A	BC	PCV13	3	17
14	6 Mar 2014	17	Meningitis	No	No	No	14	BC	PCV13	3	8
15	17 Mar 2014	19	Pneumonia	Yes	No	ND	14	BC	PCV13	2	14
16	5 Sep 2014	26	Pneumonia	No	No	ND	1	BC	PCV13	3	20
17	2 Nov 2014	24	Sepsis	Yes	No	No	19A	BC	PCV13	3	17

Supplementary Figures

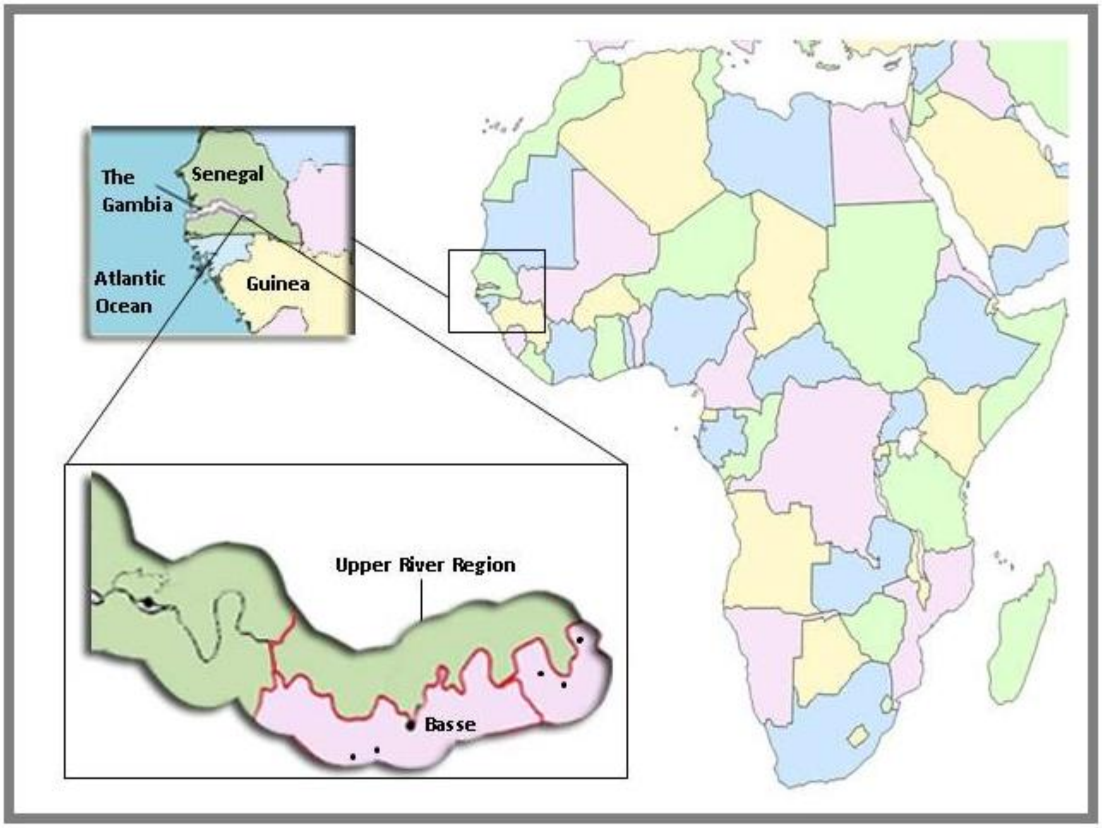

Basse Health and Demographic Surveillance System shaded pink in the inset box.

Figure S1. Map showing the location of the Basse Health and Demographic Surveillance System in a rural area of The Gambia, West Africa with the position of study health facilities shown in the insert

Figure S2. Profile of patient screening, enrolment, and investigation

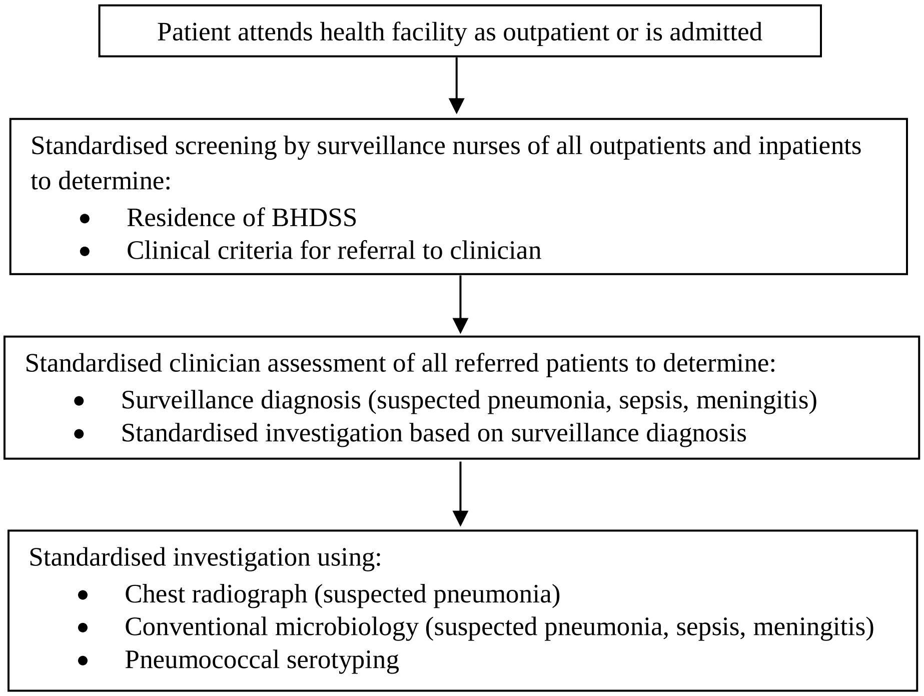

Figure S3. Proportion of patients with suspected pneumonia, septicaemia, or meningitis who had lung aspirate sample collection by year of the study

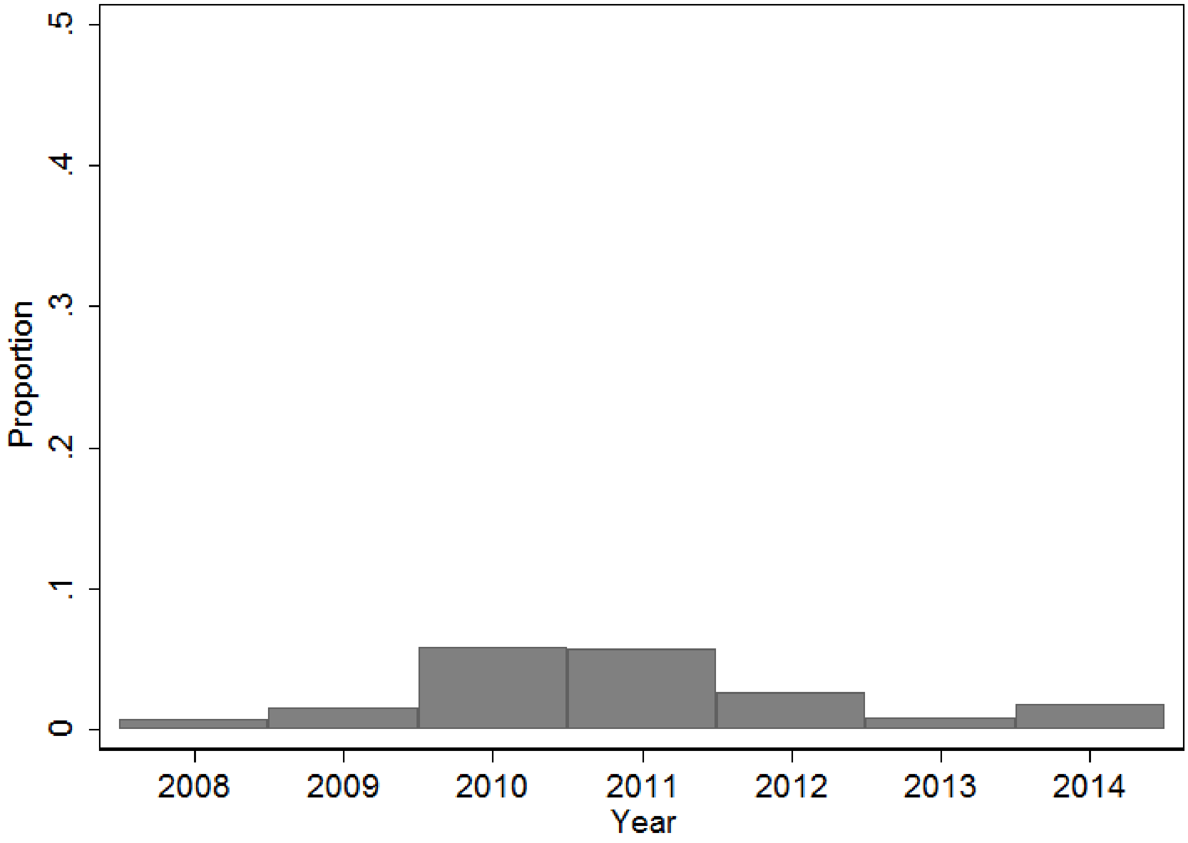

Figure S4. Coverage of two or more doses of A) PCV7 and B) PCV13 in age groups 2-5 months and 6-23 months, over time

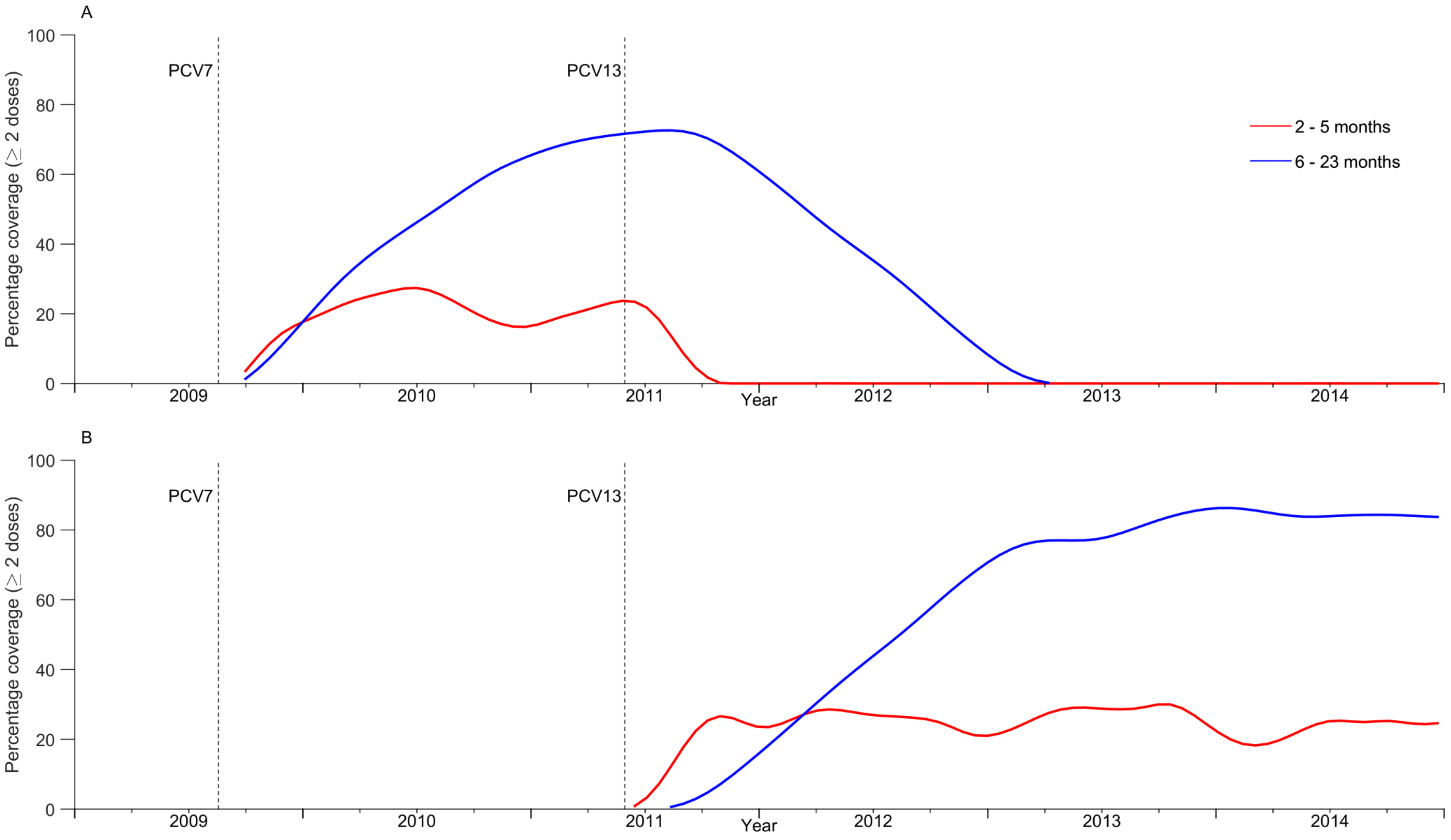

Figure S5. Adjusted incidence of non-pneumococcal bacteraemia between 2008 and 2014, by age group

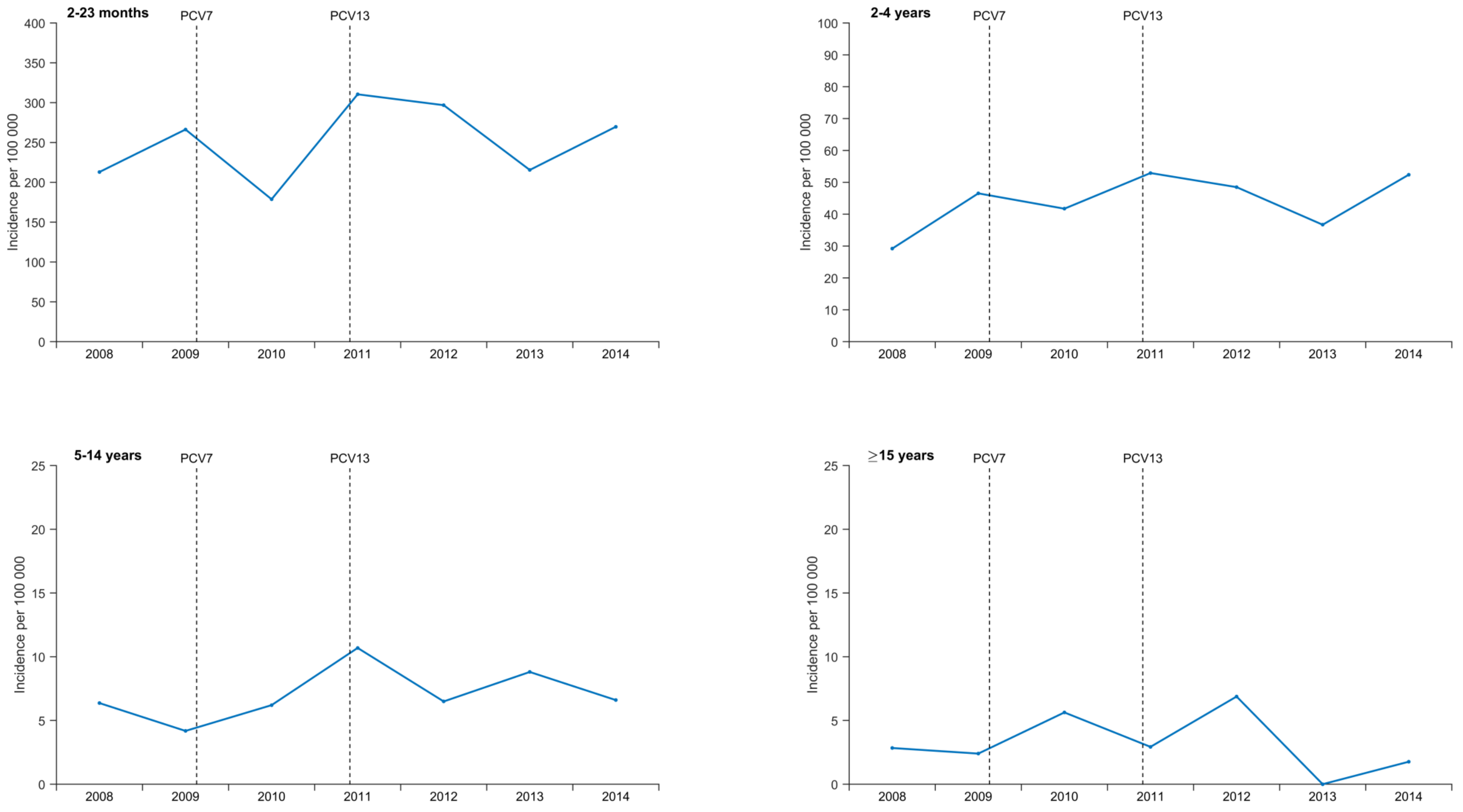

Figure S6. Proportion of children aged 2-59 months with suspected pneumonia, septicaemia, or meningitis in the wet season who had a positive malaria test by year of the study

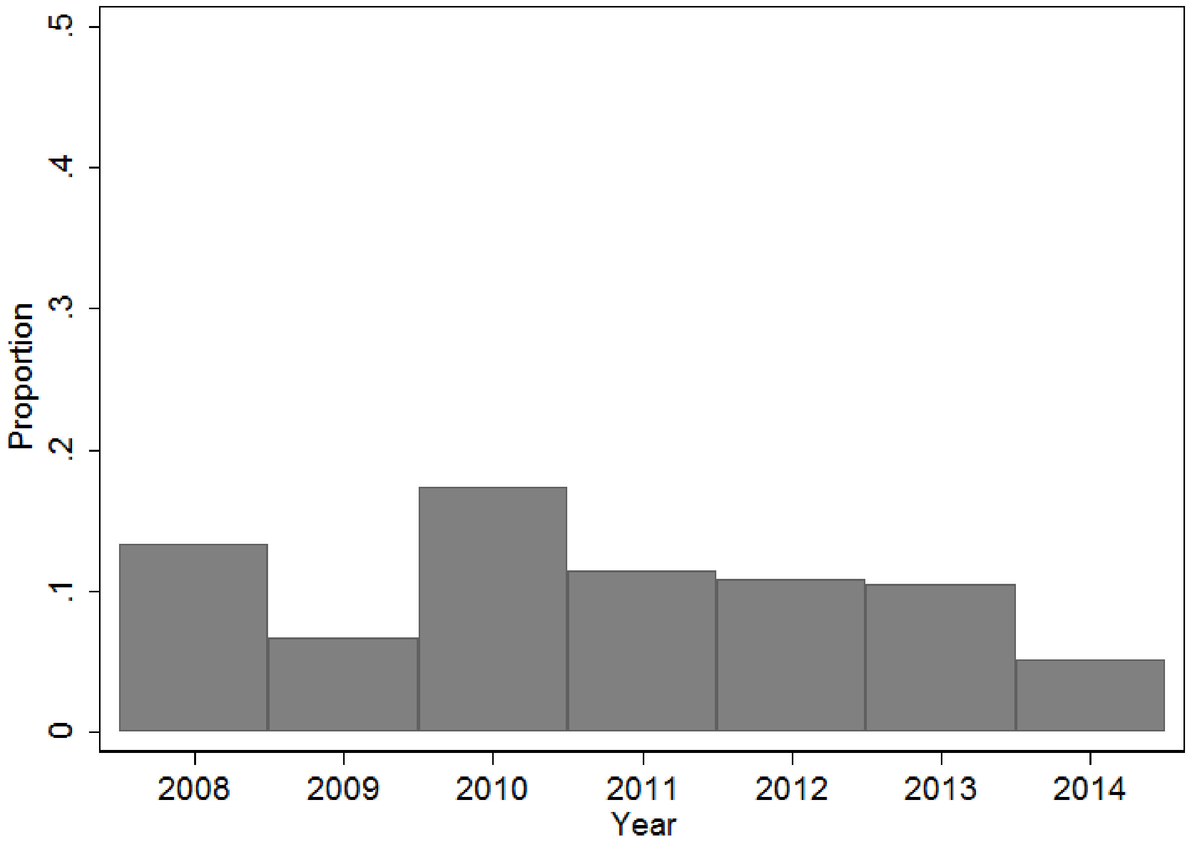

Note: the prevalence of malaria in study participants aged 2-59 months in the ‘baseline’ period and 2013/14 period was not significantly different.

Figure S7. Proportion of patients aged 5 years and greater with suspected pneumonia, septicaemia, or meningitis in the wet season who had a positive malaria test by year of the study

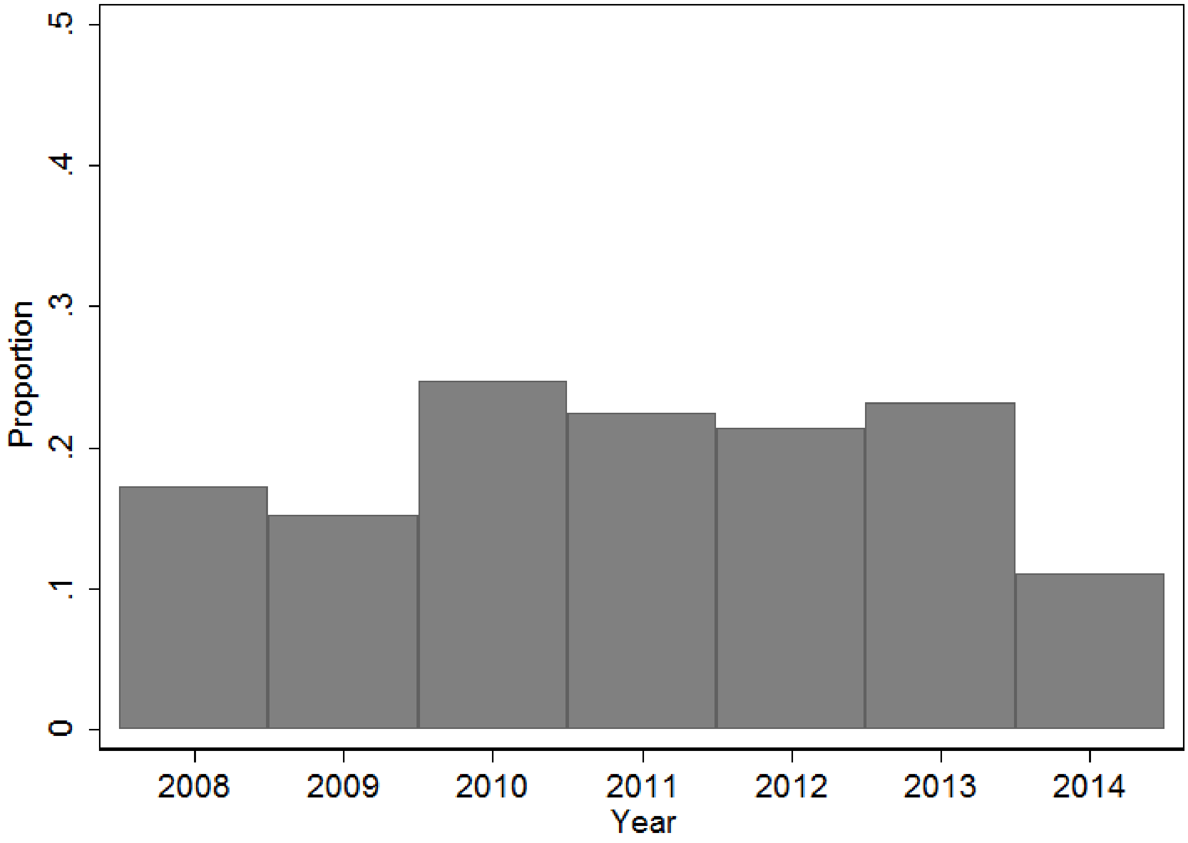

Note: the prevalence of malaria in study participants aged 5 years and greater in the ‘baseline’ period and 2013/14 period was not significantly different.

Figure S8. Proportion of children with suspected pneumonia, septicaemia, or meningitis who had weight-for-height z-score < -3 by year of the study

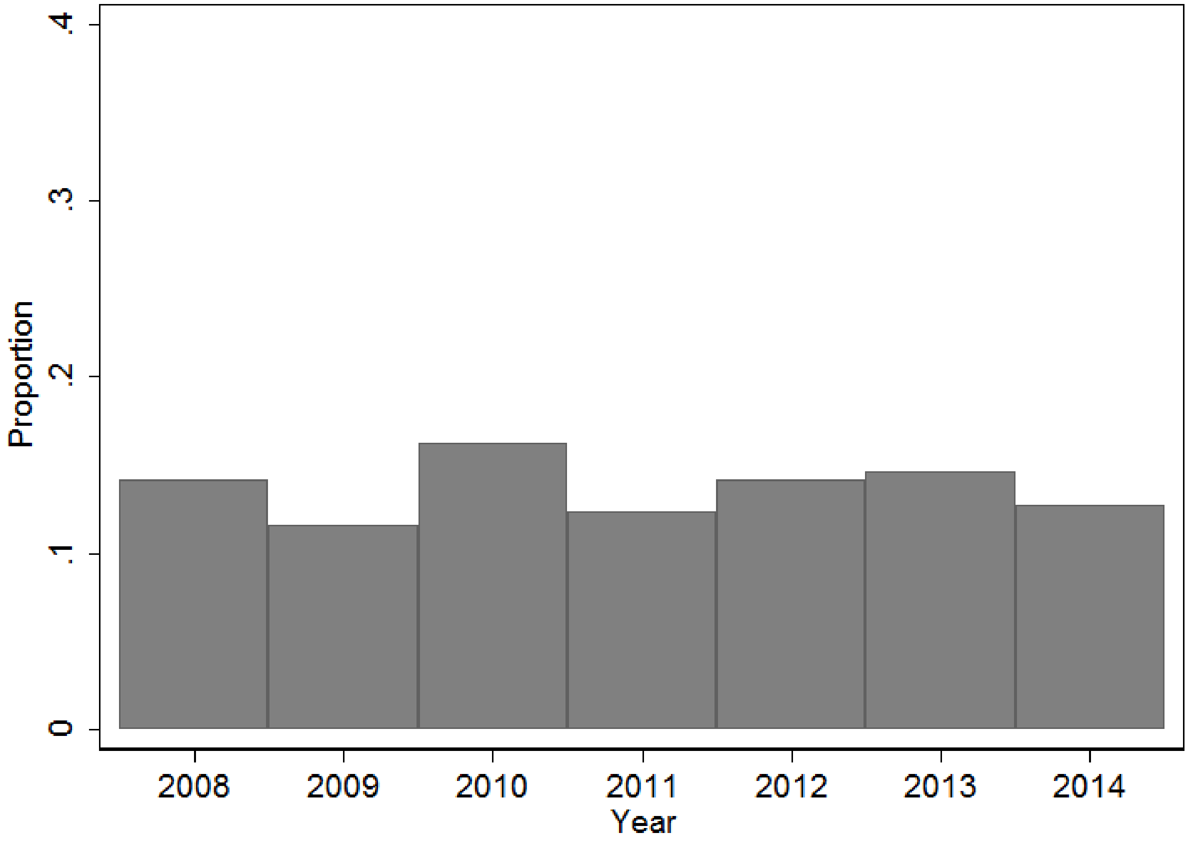

Figure S9. Proportion of children with suspected pneumonia, septicaemia, or meningitis who had contaminated blood cultures by year of the study

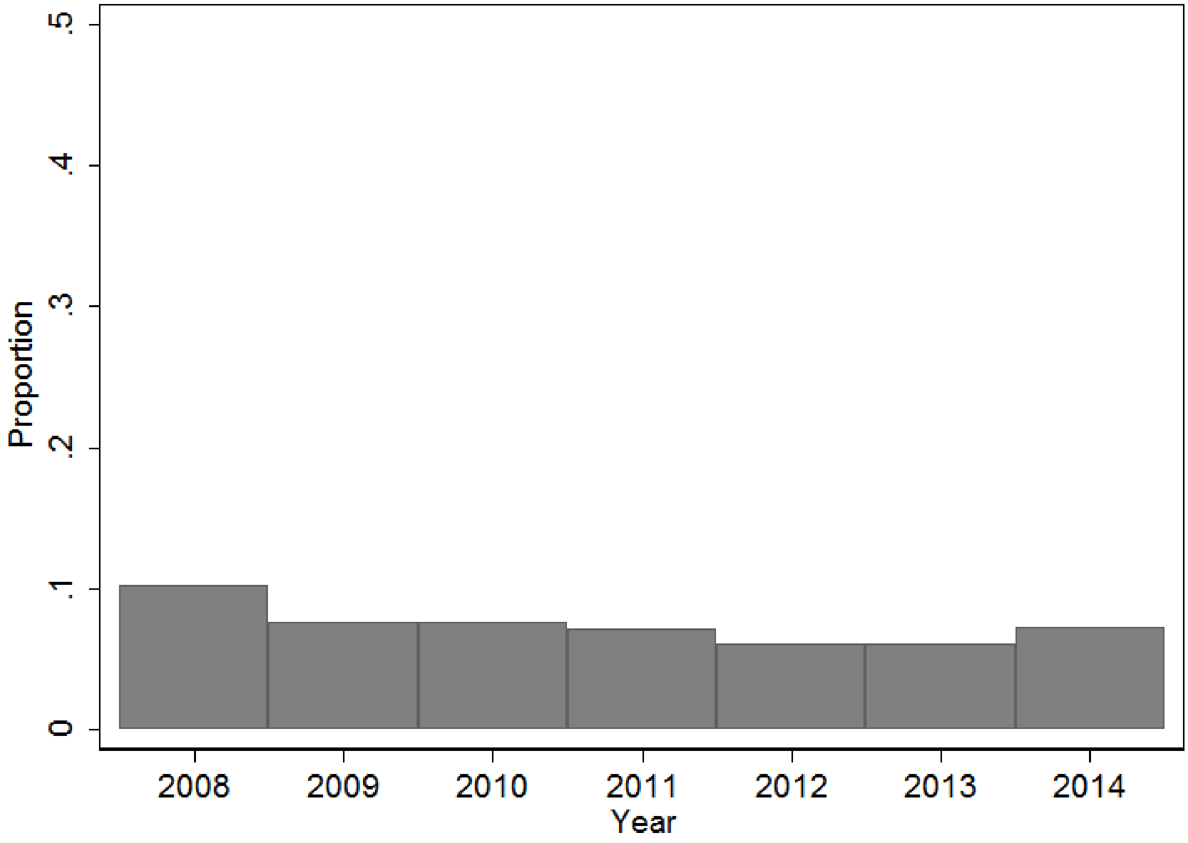

Note: the prevalence of contaminated blood cultures in study participants aged 2-59 months in the 'baseline' period and 2013/14 period was not significantly different.
